# Celastrol Downmodulates Alpha-Synuclein-Specific T Cell Responses by Mediating Antigen Trafficking in Dendritic Cells

**DOI:** 10.3389/fimmu.2022.833515

**Published:** 2022-03-02

**Authors:** Lam Ng, Xiaohui Wang, Chuanbin Yang, Chengfu Su, Min Li, Allen Ka Loon Cheung

**Affiliations:** ^1^ Department of Biology, Faculty of Science, Hong Kong Baptist University, Kowloon Tong, Hong Kong SAR, China; ^2^ Mr. & Mrs. Ko Chi Ming Center for Parkinson Disease Research, School of Chinese Medicine, Hong Kong Baptist University, Kowloon Tong, Hong Kong SAR, China; ^3^ Department of Geriatrics, Shenzhen People’s Hospital (The Second Clinical Medical College, Jinan University; The First Affiliated Hospital, Southern University of Science and Technology), Shenzhen, China; ^4^ College of Pharmacy, Henan University of Chinese Medicine, Zhengzhou, China

**Keywords:** Celastrol, Parkinson’s Disease, α-synuclein, dendritic cell, CD4^+^ T cell subsets, endo-lysosomal pathway, autophagy

## Abstract

Parkinson’s Disease (PD) is a neurodegenerative disease that affects the elderly. It is associated with motor dysfunction due to the accumulation of misfolded or aggregated fibrillar alpha-synuclein (α-syn) in the mid-brain. Current treatments are mainly focused on relieving the symptoms but are accompanied by side effects and are limited in halting disease progression. Increasing evidence points to peripheral immune cells underlying disease development, especially T cells contributing to α-syn-related neuroinflammation in PD. The onset of these cells is likely mediated by dendritic cells (DCs), whose role in α-syn-specific responses remain less studied. Moreover, Traditional Chinese medicine (TCM)-derived compounds that are candidates to treat PD may alleviate DC-T cell-mediated immune responses. Therefore, our study focused on the role of DC in response to fibrillar α-syn and subsequent induction of antigen-specific T cell responses, and the effect of TCM Curcumin-analog C1 and *Tripterygium wilfordii* Hook F-derived Celastrol. We found that although fibrillar α-syn did not induce significant inflammatory or T cell-mediating cytokines, robust pro-inflammatory T cell responses were found by co-culturing fibrillar α-syn-pulsed DCs with α-syn-specific CD4^+^ T cells. Celastrol, but not C1, reduced the onset of pro-inflammatory T cell differentiation, through promoting interaction of endosomal, amphisomal, and autophagic vesicles with fibrillar α-syn, which likely lead to its degradation and less antigen peptides available for presentation and T cell recognition. In conclusion, regulating the intracellular trafficking/processing of α-syn by DCs can be a potential approach to control the progression of PD, in which Celastrol is a potential candidate to accomplish this.

## Introduction

Parkinson’s Disease (PD) is the second most common neurodegenerative disease in the world, affecting approximately 10 million people worldwide who are aged over 60 (1). PD is characterized by impaired motor symptoms due to the progressive loss of functions or cell death of dopaminergic neurons in the par compacta of substantia nigra (SNpc) or caudate putamen (CPu) of the brain (2). α-Synuclein (α-syn) is a presynaptic protein that is expressed abundantly in the mammalian brain and peripheral tissues (3). Under normal physiological conditions, it exists in its intrinsic unfolded monomeric conformation allowing its interactions with other proteins, such as SNARE complex proteins, to perform normal cell trafficking functions (4). One pathological hallmark of PD is the accumulation of misfolded α-syn protein inclusions forming Lewy bodies inside the neuronal cells that are caused by either familial inherited gene mutations or idiopathic factors (5–7). The abnormal α-syn in the shape of the β-sheet structure serve as nucleation sites, which further recruit endogenous monomeric α-syn, convert into soluble oligomeric form that at a later stage, aggregate into insoluble fibrillar form (8, 9). It is proposed that accumulation of oligomeric or fibrillar α-syn leads to dopaminergic neuronal cell death and activates the brain resident microglia causing neuroinflammation (10, 11). Dysregulated and prolonged neuroinflammation will lead to more neuronal cell death and compromise the integrity of the blood–brain barrier (BBB), favor the recruitment and infiltration of peripheral immune cells which could worsen the disease (12–14).

In PD patients, T cells were found in the brain parenchyma and the relative abundance of T cells in peripheral blood was decreased compared to healthy control (12, 15). Despite this, the frequency of Th1, Th17 but not Treg subsets were elevated in PD patients and associated with the increased serum levels of IL-6 and IL-17 and decreased IL-10 and TGF-β (16, 17). The polarization of these subsets is governed by cytokines. Th1 differentiation is dependent on IL-12, IL-18, and IFN-γ that trigger T-box transcription factor TBX21 (T-bet) transcription factor; Th17 uses retinoic acid receptor-related orphan receptor gamma t (RORγt) transcription factor induced by IL-1β, IL-6, IL-23, and TGF-β; Treg differentiation depends on forkhead box P3 (FoxP3) stimulated by IL-10, IL-2, and TGF-β (18–21). In PD patients, these T cells can recognize α-syn peptides even before the onset of motor symptoms (22, 23). Moreover, brain neurons that express IL-17 receptors are prone to neuronal cell death driven by IL-17 and NFκB activation (24).

DCs provide the three signals for CD4^+^ T cell activation and differentiation, through major histocompatibility complex class II (MHC-II) antigen presentation (signal 1), co-stimulatory molecules (signal 2), and cytokines (signal 3) (25, 26). Normally, absence or low abundance of peripheral DCs are found in the central nervous system (CNS). In PD, peripheral DCs could migrate across the BBB and uptake the α-syn aggregates in the brain, then proceed to the cervical lymph nodes, where they increase the expression of MHC-II and present the α-syn antigenic peptides to activate antigen-specific T cells (13, 27, 28). Previous studies have demonstrated that the frequency of DCs in the blood of PD patients declined but increased in the CNS, where they could respond to α-syn aggregates and trigger neuroinflammation (29, 30). However, it remains controversial whether DCs mature in response to α-syn. DCs treated with α-syn resulted in an upregulation in the expression of co-stimulatory molecules CD80 and CD86 and also MHC-II (31), while others found no differences in the activation markers for DCs between PD patients and healthy control (32).

Endo-lysosomal degradation of foreign antigens internalized by antigen presenting cells (APCs) has long been reported for antigen presentation and mediating T cell immunity (33–35). Also, autophagy was shown to play a vital role in degrading protein aggregates for antigen presentation, which is inhibited by α-syn fibrils (36, 37). There is evidence on the linkage between autophagy gene mutations and the accumulation of α-syn (38). Moreover, mutation of autophagy-related Leucine-rich repeat kinase 2 (LRRK2) could promote DC antigen presentation to CD4^+^ T cells (39). Furthermore, α-syn can escape from lysosomal degradation by rupturing endosomal and lysosomal vesicles and resulting in increased reactive oxygen species (ROS) and inflammasome activation (40). It is reported that the limited proteolytic reaction also favors MHC class II-peptide loading and presentation to CD4^+^ T cells (41). Ras-related protein in brain (Rab) proteins are important in mediating vesicular trafficking for endocytosis, autophagy, and lysosomal degradation. They are characterized with respect to their intracellular localizations and trafficking functions. Usually, Rab5 and Rab14 (early endosome markers), Rab7 (late endosome marker), Rab11 (recycling endosome marker), and Rab9 and Lamp1 (lysosome markers), form the different stages of the endo-lysosomal pathway (42). In addition, Rab proteins play a role in autophagy by interacting with Beclin1 and LC3 (43, 44), which may be related to the trafficking of α-syn for antigen processing and presentation in DCs. To assist in defining the importance of these pathways, we used two traditional Chinese medicine (TCM)-derived compounds which are previously shown as potential autophagy inducers: C1—a novel Curcumin analog (45), and Celastrol—a natural bioactive compound derived from *Tripterygium wilfordii* Hook F (TWHF) (46, 47), where the latter also displayed anti-inflammatory effect in different diseases (48, 49). Though, whether these drugs have any effects against α-syn-specific T cell immune response have not been investigated. Therefore, we hypothesized that Celastrol and C1 could decrease DC-mediated α-syn-specific pro-inflammatory T cell responses by modulating the trafficking pathways that could possibly favor the processing of α-syn leading to reduced T cell activation.

In this study, we addressed three main issues: 1) Monocyte-derived DCs (MoDCs) response toward α-syn stimulation; 2) CD4^+^ T cell subsets abundance in response to α-syn-pulsed MoDCs; and, 3) the interactions of endo-lysosomal and autophagic pathway components with α-syn in MoDCs. Interestingly, our data showed that α-syn treatment did not induce higher expression of antigen presentation molecules nor cytokines gene transcription in MoDCs, but still stimulated robust inflammatory α-syn specific CD4^+^ T cell responses. Both endo-lysosomal and autophagic pathways are associated with α-syn, where colocalizations of Rab and autophagosomal proteins with α-syn were found. Treatment of MoDCs with α-syn appeared to downregulate the expression of Rab and autophagosomal proteins, indicating the possibility that α-syn regulated its interaction with antigen processing components which limited its degradation and allowed the activation of T cells. Lastly, Celastrol (but not C1) could inhibit α-syn-specific T cell responses, likely through the induction of higher colocalization of α-syn with Rab5^+^ and Rab7^+^ vesicles and autophagosomes, which suggest a more effective degradation that may assist in minimizing antigen presentation to CD4^+^ T cells.

## Materials and Methods

### CD14^+^ Monocytes Isolation and Differentiation Into MoDCs

Peripheral blood mononuclear cells (PBMCs) were freshly isolated from the whole blood of anonymous healthy human blood donors using Lymphoprep (Cat. No. 07851, Stemcell Technologies). Usage of healthy human blood received approval from the HKBU Research Ethics Committee (#REC/19-20/0110). CD14^+^ monocytes were isolated from the freshly prepared PBMCs using CD14 microbeads, human (Cat. No. 130-050-201, Miltenyi Biotec) according to manufacturer protocol. To generate MoDCs, CD14^+^ cells were cultured at a density of 1 × 10^6^ cells/ml in RPMI 1640 medium (Cat. No. 11875-085, Gibco) supplemented with 10% Fetal Bovine Serum (FBS) (Cat. No. 10270-106, Gibco), 1% Penicillin–Streptomycin (10,000 U/ml) (Cat. No. 15140-122, Gibco), 25 µg/ml rhIL-4 (Cat. No. 200-04, PeproTech), and 25 µg/ml rhGM-CSF (Cat. No. 300-03, PeproTech) for 5–7 days, with 50% medium change every 3 days. Cells were incubated at 37°C, 5% CO_2_.

### Reverse Transcription Quantitative Real-Time PCR (qRT-PCR)

MoDCs were seeded at a density of 1 × 10^6^ cells/ml in RPMI 1640 medium 1% FBS in 24-well cell culture plate overnight at 37°C, 5% CO_2_. C1 (1 µM) and Celastrol (0.25 µM) were added to pre-treat MoDCs for 1 h and recombinant Human Alpha-synuclein protein aggregate (Active) (fibrillar α-syn) (Cat. No. ab218819, Abcam) (1 µg/ml) was used to treat MoDCs for another 4 h. Cells were harvested, and total RNA was extracted using RNAiso Plus followed by cDNA generation by PrimeScript™ RT Master Mix (Cat. No. RR047A, Takara). Quantitative real-time PCR was carried out with the TB Green Premix Ex Taq II (Tli RNase H Plus) (Cat. No. RR820A, Takara) using StepOnePlus™ Real Time System (Cat. No. 4376600, Invitrogen). The following primer pairs were used: GAPDH, 5′-ACAGTCCATGCCATCACTGCC-3′, 5′-GCCTGCTTCACCACCTTCTTG-3′; IL-1β, 5′-ATGATGGCTTATTACAGTGGCAA-3′, 5′-GTCGGAGATTCGTAGCTGGA-3′; IL-6, 5′-AGACAGCCACTCACCTCTTC-3′, 5′-AGTGCCTCTTTGCTGCTTTC-3′; IL-23, 5′-TTTTCACAGGGGAGCCTTCT-3′, 5′-ACTGAGGCTTGGAATCTGCT-3′; TNF-α, 5′-GTCAACCTCCTCTCTGCCAT-3′, 5′-CCAAAGTAGACCTGCCCAGA-3′; TGF-β, 5′-CACGTGGAGCTGTACCAGAA-3′, 5′-GAACCCGTTGATGTCCACTT-3′; IL-10, 5′-GACTTTAAGGGTTACCTGGGTTG-3′. 5′-TCACATGCGCCTTGATGTCTG-3′. Relative expression was calculated by normalizing to GAPDH and by ΔΔCT method. The ΔΔCT method was used to calculate the relative expression of each gene with reference to GAPDH, which were then normalized to α-syn only treatment.

### BV2 Culturing and Treatment

Mouse BV2 microglia cell line was purchased from Elabscience (Cat. No. EP-CL-0493) and cultured in DMEM, high glucose (Cat. No. 11965-126, Gibco) supplemented with 10% FBS. Cells were incubated at 37 °C, 5% CO_2_ and subcultured when cell confluency reached 80–90%. Before treatment, BV2 were seeded in DMEM medium with 10% FBS in 24-well cell culture plate overnight at 37 °C, 5% CO_2_. The next day, lipopolysaccharide (LPS) (100 ng/ml) was used to prime the cells for 3 h in DMEM medium with 10% FBS and washed 3 times with PBS and replaced with fresh DMEM medium. Then, fibrillar α-syn in a concentration of 1 µg/ml was used to treat the BV2 cells for 3 h. Cells were harvested and subjected to qRT-PCR using similar methods as above.

### Flow Cytometry for MoDC Surface Markers

MoDCs were seeded at a density of 1 × 10^5^ cells/ml in RPMI 1640 medium 1% FBS in 24-well cell culture plate overnight at 37°C, 5% CO_2_. C1 (1 µM) and Celastrol (0.25 µM) were added to pre-treat MoDCs for 1 h followed by the addition of fibrillar α-syn (1 µg/ml) for 24 h. In some cases, TNF-α (50 ng/ml) was added together with fibrillar α-syn. LPS (100 ng/ml) was used as the positive control. Afterward, MoDCs were collected and washed with fluorescence-activated cell sorter (FACS) buffer (PBS + 1% FBS) and incubated for 30 min at 4°C in 100 µl FACS Buffer with BV421 Mouse anti-Human HLA-ABC (Cat. No. 565332, BD Pharmingen), APC Mouse anti-Human HLA-DR (Cat. No. 560896, BD Pharmingen), PE-Cy7 Mouse anti-Human CD86 (Cat. No. 561128, BD Pharmingen) and FITC Mouse anti-Human CD80 (Cat. No. 555683, BD Pharmingen) antibodies. Flow cytometry was performed following standard protocols on a BD FACSCanto™ II cytometer.

### MoDC Stimulation of Antigen-Specific CD4^+^ T Cells

α-Syn peptide-specific CD4^+^ T cells were generated according to the previous protocol (50) with some modifications. Briefly, freshly isolated PBMCs (5 × 10^6^ cells/ml) were first cultured in RPMI + 10% FBS + 1% GlutaMAX (Cat. No. 35050-061, Gibco) and stimulated with α-syn peptide EQVTNVGGAVVTGVT (5 µg/ml) (ChinaPeptides) with reference to previous studies (22). One day after stimulation, rhIL-2 (100 IU/ml) (Cat. No. 200-02, Preprotech) was added to the cell culture for 7 days, with 50% media change and replenishment of rhIL-2 every 3 days. Frozen autologous PBMCs were stimulated with the same α-syn peptide (5 µg/ml) overnight and were added into the original PBMCs culture in a ratio of 1:10 for re-stimulation for another 7 days, with 50% media change and replenishment of rhIL-2 every 3 days. Simultaneously, MoDCs were generated as mentioned above and cultured for 6 days. Prior to the co-culture experiment, MoDCs were pre-treated with either C1 (1 µM) or Celastrol (0.25 µM) for 1 h followed by treating with fibrillar α-syn (1 µg/ml) overnight in RPMI + 10% FBS + 1% GlutaMAX. Also, rhIL-2 concentration of the PBMCs culture was reduced to 20 IU/ml to minimize its effect on T cells but to maintain survival. On the day of the co-culturing experiment, total CD4^+^ T cells were isolated from the PBMCs culture using CD4^+^ T Cell Isolation Kit, human (Cat. No. 130-096-533, Miltenyi Biotec) and added into the MoDCs culture in a ratio of MoDCs:T cell = 1:5 and cultured for 1 day or 3 days. α-Syn-specific CD4^+^ T cell only was served as the negative control. One day after the co-culture experiment, half of the suspension cells were harvested for flow cytometry analysis of surface CD3, CD4, CD25, and intracellular IFN-γ and T-bet expression using FITC Mouse anti-Human CD3 (Cat. No. 555339, BD Pharmingen), V500 Mouse anti-Human CD4 (Cat. No. 560768, BD Pharmingen), APC-Cy7 Mouse anti-Human CD25 (Cat. No. 557753, BD Pharmingen), PE-Cy7 Mouse anti-Human IFN-γ (Cat. No. 557643, BD Pharmingen) and PerCP-Cy5.5 Mouse anti-Human T-bet (Cat. No. 561316, BD Pharmingen) antibodies. While on day 3 of the co-culturing experiment, the remaining suspension cells were harvested for flow cytometry analysis of surface CD3, CD4, CD25, and intracellular IL-17A, RORγt and FoxP3 expression using FITC Mouse anti-Human CD3 (Cat. No. 555339, BD Pharmingen), V500 Mouse anti-Human CD4 (Cat. No. 560768, BD Pharmingen), APC-Cy7 Mouse anti-Human CD25 (Cat. No. 557753, BD Pharmingen), Alexa Fluor 647 Mouse anti-Human RORγt (Cat. No. 563620, BD Pharmingen), PerCp-Cy5.5 Mouse anti-Human IL-17A (Cat. No. 560799, BD Pharmingen) and PE Mouse anti-Human FoxP3 (Cat. No. 560046, BD Pharmingen) antibodies. Flow cytometry was performed following standard protocols on a BD FACSCanto™ II cytometer.

### Immunofluorescence Analysis of Rab and Autophagy-Related Proteins Using Confocal Microscopy

MoDCs (1 × 10^5^ cells/200 µl) were seeded on Nunc™ Lab-Tek™ II 8-well Chambered Coverglass w/non-removable wells (1.5 Borosilicate Glass) (Cat. No. 155409, Invitrogen) in RPMI with 1% FBS overnight at 37 °C, 5% CO_2_. For studying endo-lysosomal pathway, C1 (1 µM) and Celastrol (0.25 µM) were added to pre-treat MoDCs for 1 h followed by the addition of 1 µg/ml fibrillar α-syn for 15, 30, and 60 min. Cells were then fixed with 2% paraformaldehyde (PFA) for 20 min at room temperature, permeabilized using the blocking and permeabilizing buffer composed of PBS with 5% Normal Goat Serum (Cat. No. 31873, Invitrogen), 3% Bovine Serum Albumin (BSA) (Cat. No. 9048-46-8, Sigma) and 0.5% Triton X-100 for 20 min, 4 °C. Following washing, primary antibodies were used for overnight incubation in the staining buffer of PBS with 3% BSA and 0.1% Triton X-100 overnight at 4°C: for early endosome markers, Mouse anti-Rab5 (1:200) (Cat. No. ab66746, Abcam) and Rabbit anti-Rab14 (1:200) (Cat. No. ab40938, Abcam) were incubated with 15 min samples; late endosome marker, Mouse anti-Rab7 (1:500) (Cat. No. ab50533, Abcam) and recycling endosome maker Rabbit anti-Rab11 (1:500) (Cat. No. ab36112, Abcam) were incubated with 30 min samples; lysosome markers, Mouse anti-Rab9 (1:500) (Cat. No. MA3-067, Invitrogen) and Rabbit anti-Lamp1 (1:300) (Cat. No. ab24170, Abcam) were incubated with 60 min samples. All samples were also probed with Chicken anti-α-syn (1:1,000) (Cat. No. ARG10689, Arigobio) in the staining buffer of PBS with 3% BSA and 0.1% Triton X-100 overnight at 4 °C.

For studying autophagic pathways, after C1 or Celastrol pre-treatment, MoDCs were treated with 1 µg/ml fibrillar α-syn for 4, 6 or 16 h. Cells were then fixed and permeabilized as mentioned above. Primary antibodies of early autophagosome marker, Rabbit anti-Beclin1 (1:200) (Cat. No. ab62557, Abcam) and early endosome marker, Mouse anti-Rab5 (1:500) were incubated with 4 h samples; autophagosome cargo protein p62 (SQSTM1), Mouse anti-SQSTM1/p62 (1:200) (Cat. No. ab56416, Abcam) was incubated with 6 h samples; autophagosome marker, Rabbit anti-LC3B (1:300) (Cat. No. NB100-2220, Novus Biologicals) and late endosome marker, Mouse anti-Rab7 (1:500) were incubated with 16 h samples. All samples were also probed with Chicken anti-α-syn (1:1,000) in the staining buffer of PBS with 3% BSA and 0.1% Triton X-100 overnight at 4°C.

Following primary antibody staining, cells were washed 3 times with PBS + 0.01% Triton X-100. Secondary antibodies Alexa Fluor 488 Goat anti-Rabbit (1:1,000) (Cat. No. A27034, Invitrogen), Alexa Fluor 647 Goat anti-Mouse (1:1,000) (Cat. No. A28181, Invitrogen) and DyLight anti-Chicken IgY H&L (1:500) (Cat. No. ab96948, Abcam) were used for fluorescence staining at room temperature for 1 h in darkness. Cells were washed 3 times again with PBS with 0.01% Triton X-100. Cell nuclei were stained with Hoechst 33258 (1:800) (Cat. No. ab228550, Abcam) for 20 min at 4 °C in darkness. Lastly, cells were mounted using ibidi mounting medium (Cat. No. 50001, ibidi) and signals were acquired using Leica TCS SP5 confocal microscopy and images were analyzed using the LAS AF software (Leica). The number of Rab5, Rab7, Rab9, Rab11, Rab14, Beclin1, LC3 positive puncta and their colocalizations with α-syn were counted and colocalization intensity values were analyzed using ImageJ software (http://imagej.nih.gov/ij/) (51).

### Western Blot

Cells were collected and washed with PBS, followed by lysing with lysis buffer (10 mM Tris–HCl (pH 7.5), 200 mM NaCl, 1 mM EDTA, 1 mM DTT, 0.5% NP-40, 10 µg/ml aprotinin, 10 µg/ml leupeptin, 1.25 µg/ml pepstatin A, 1 mM PMSF) on ice for 45 min. Lysates were then centrifuged at 13,000×*g*, 4°C for 30–45 min. Supernatants were collected and protein concentration was measured using Pierce™ BCA Protein Assay Kit (Cat. No. 23225, Thermo Scientific) according to the manufacturer’s instructions. Protein samples were mixed with 5× loading buffer (250 mM Tris–HCl (pH 6.8), 10% SDS, 30% glycerol, 5% β-mercaptoethanol, 0.02% bromophenol blue) and were boiled at 98°C for 5 min before being loaded into 10–12% SDS-PAGE gel for electrophoresis and transferred to Immobilon^®^-P polyvinylidene difluoride (PVDF) (Cat. No. P2938, Sigma) membrane using a Mini Trans-Blot Electrophoretic Transfer Cell (Bio-Rad). Membranes were blocked with 0.5% BSA and 5% blotting-grade blocker (Biorad) in PBS-T or TBS-T for 1 h at room temperature and blotted with aforementioned primary antibodies Rabbit anti-Rab5, Mouse anti-Rab7, Rabbit anti-Beclin1, Mouse anti-SQSTM1/p62, Rabbit anti-LC3B and anti-beta-Actin, clone RM112 monoclonal antibody (Cat. No. MABT523, Merck Millipore) (1:500–1:1,000) overnight at 4°C, followed by incubating with secondary antibodies HRP conjugate Donkey Anti-Rabbit antibody (Cat. No. AP182P, Merck Millipore) or HRP conjugated Goat Anti-Mouse antibody (Cat. No. AP130P, Merck Millipore) (1:10,000) for 1 h at room temperature. Membrane blots were developed using SuperSignal™ West Pico PLUS Chemiluminescent Substrate (Cat. No. 34580, Thermo Scientific) or SuperSignal™ West Femto Maximum Sensitivity Substrate (Cat. No. 34094, Thermo Scientific). Signals were detected by ChemiDoc (Bio-Rad) and band intensities were quantified using ImageJ software.

### Immunoprecipitation

Immunoprecipitation of Rab5-GTP or Rab7-GTP from MoDCs using (Cat. No. 83701, NewEast Biosciences) and Anti-Active Rab7 Mouse Monoclonal Antibody (Cat. No. 26923, NewEast Biosciences) according to the manufacturer protocol with some modifications. Briefly, cells were lysed using 1× Assay/Lysis Buffer with 10 µg/ml aprotinin, 10 µg/ml leupeptin, 1.25 µg/ml pepstatin A, 1 mM PMSF for 30–45 min on ice. Lysates were centrifuged at 13,000×*g*, 4°C for 30–45 min. Supernatants were collected and protein concentration was measured using Pierce™ BCA Protein Assay Kit according to manufacturer’s instructions. Afterward, lysates were incubated with either Mouse anti-Rab5-GTP or Mouse anti-Rab7-GTP primary antibody (1 µg) pre-conjugated with protein A/G agarose slurry (20 µl) in 500 µl of 1× Assay/Lysis Buffer with protease inhibitors for a maximum of 2 h, 4°C, with gentle rotation for immunoprecipitation. Afterward, 5× loading buffer was used to elute the targeted proteins by boiling at 98°C for 5 min and the protein lysates were subjected to SDS-PAGE as described above. Membranes were blocked with TBS-T + 3% BSA and incubated with either Rabbit anti-Rab5 or Mouse anti-Rab7 primary antibody (1:1,000) in TBS-T + 3% BSA overnight at 4°C, followed by incubating with Recombinant Protein G (HRP) Cat. No. (ab7460, Abcam) (1:1,000) in TBS-T + 3% BSA for 1 h at room temperature. Membrane blots were developed using SuperSignal™ West Pico PLUS Chemiluminescent Substrate or SuperSignal™ West Femto Maximum Sensitivity Substrate. Signals were detected by ChemiDoc (Bio-Rad) and quantified using ImageJ software. A total of 25 µg of total cell lysates were used as input control.

### Analysis of C1 and Celastrol Potential Targets in α-Syn Antigen Trafficking and Presentation Using Online Tools

The putative protein targets associated with C1 and Celastrol were predicted using SwissTargetPrediction tool (http://www.swisstargetprediction.ch). The protein–protein interaction networks of the predicted targets were generated using the STRING database (http://www.string-db.org/) (52). Potential proteins and pathways that are related to antigen processing and presentation were identified and grouped. Details of the analysis can be found in **Supplementary Figures 10–12** and **Tables S1, S2**.

### Statistical Analysis

Data generated were from at least three independent experiments. Results were presented as the mean ± standard deviation (SD), unless specified. Statistical significance was calculated by the One-way ANOVA and Tukey’s multiple comparisons test were used to analyze differences among treatment groups, unless specified. A probability value of *P <*0.05 was considered statistically significant.

## Results

### MoDCs Activation and Inflammatory Responses Towards Fibrillar α-Syn

**Figure 1:**
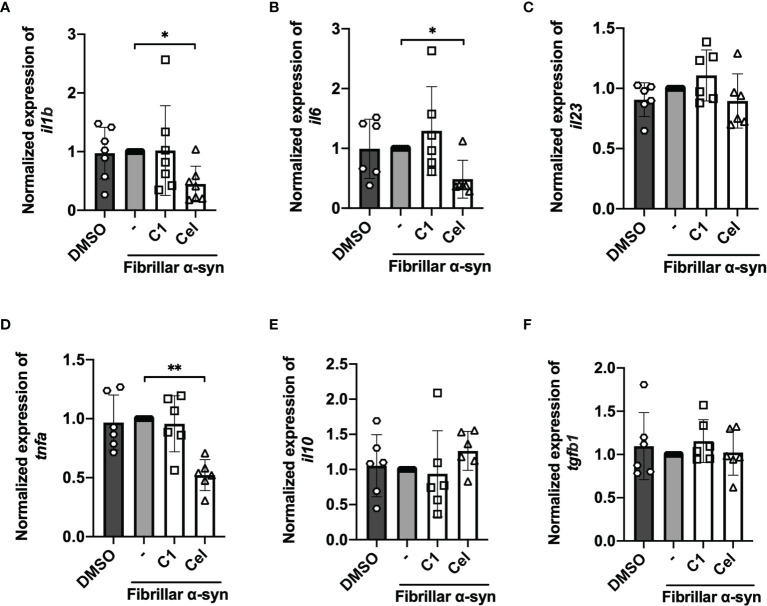
Inflammatory cytokine gene expression of fibrillar α-syn-treated MoDCs. MoDCs were pre-treated with C1 (1 µM) or Celastrol (0.25 µM) for 1 h followed by treating with fibrillar α-syn (1 µg/ml) for 4 h. Relative qRT-PCR measured gene expression compared to GAPDH were calculated and were then normalized to α-syn only treatment. **(A)** *il1b*, **(B)** *il6*, **(C)** *il23*, **(D)** *tnfa*, **(E)** *il10*, and **(F)** *tgfb1*. Column graph data represents mean ± SD from 5 individual experiments. Statistical significance was calculated by one-way ANOVA and Tukey’s multiple comparisons test, *P < 0.05, **P < 0.01.

To understand the role of DC in stimulating α-syn-specific T cell responses, we first treated MoDCs with fibrillar α-syn, and evaluated the effect of C1 and Celastrol by pre-treatment. The expression of antigen presenting MHC-I (or HLA-A,B,C) and MHC-II (or HLA-DR), co-stimulatory CD80 and CD86, and also the inflammatory cytokine gene expression were assessed. MoDCs were pre-treated with C1 or Celastrol for 1 h before being treated with fibrillar α-syn for another 4 h. Gene expression of pro-inflammatory cytokines IL-1β, IL-6, IL-23, TNF-α, and anti-inflammatory IL-10 and TGF-β were assessed by qRT-PCR. When compared to the untreated cells, α-syn had no significant induction of cytokine gene expression (**Figure 1**). However, treatment using the same α-syn on mouse microglia BV2 cells resulted in the upregulation of these cytokines (**Supplementary Figure 1**). Interestingly, a significant reduction of IL-1β, IL-6, and TNF-α gene expression and a slight upregulation of anti-inflammatory IL-10 gene expression were observed with Celastrol pre-treatment while C1 had no effects (**Figures 1A, B, D, E**). Our results suggested that α-syn had minimal effects on inflammatory cytokine gene expression in MoDCs, but this can still be modulated by Celastrol.

**Figure 2:**
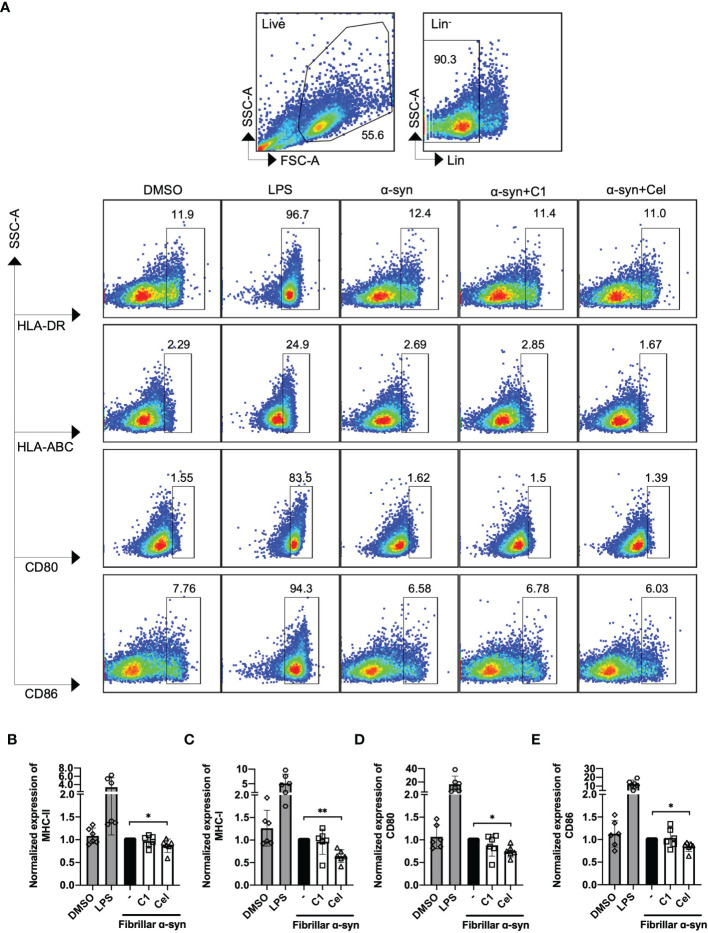
Analysis of surface expression of MHC-I/II and co-stimulatory molecules of fibrillar α-syn-treated MoDCs. MoDCs were pre-treated with C1 (1 µM) or Celastrol (0.25 µM) for 1 h followed by treatment with fibrillar α-syn (1 µg/ml) for 24 h. Surface expression of HLA-ABC, HLA-DR, CD80, and CD86 were assessed by flow cytometry. **(A)** Gating strategy and representative dot plots of HLA-DR (MHC-II), HLA-ABC (MHC-I), and CD80, CD86 expression from 6 individual experiments are shown. Live cells were first identified followed by gating lineage^-^ cells representing MoDCs. Cells were further gated as HLA-DR^+^, HLA-ABC^+^, CD80^+^, and CD86^+^ cells. DMSO as the negative control and LPS as the positive control. **(B–E)** Column graphs showing frequencies of positive cells normalized to α-syn only treatment. Column graph data represents mean ± SD from 6 individual experiments. Statistical significance was calculated by one-way ANOVA and Tukey’s multiple comparisons test, *P < 0.05, **P < 0.01.

We next determined the surface expression of MHC-I, MHC-II, CD80, and CD86 on fibrillar α-syn-treated MoDCs. After 24 h of treatment, we found that fibrillar α-syn had no significant effect on the expression of these molecules in both cell frequencies (%) or mean fluorescence intensities (MFI) when compared to the negative control (**Figure 2** and **Supplementary Figure 2**). TNF-α treated DCs can complement antigen uptake and DC maturation (53–55). In our experiments, however, though TNF-α led to upregulation of MHC-I, MHC-II, and CD86 in all treatments, the effect of subsequent α-syn stimulation appears to be negligible (**Supplementary Figure 3**). C1 and Celastrol had no effects in this experiment. Therefore, α-syn did not result in MoDCs upregulation of antigen presentation molecules, co-stimulatory molecules, or cytokines.

### α-Syn-Treated MoDC Elicited Pro-Inflammatory CD4^+^ T Cells That is Counteracted by Celastrol

**Figure 3:**
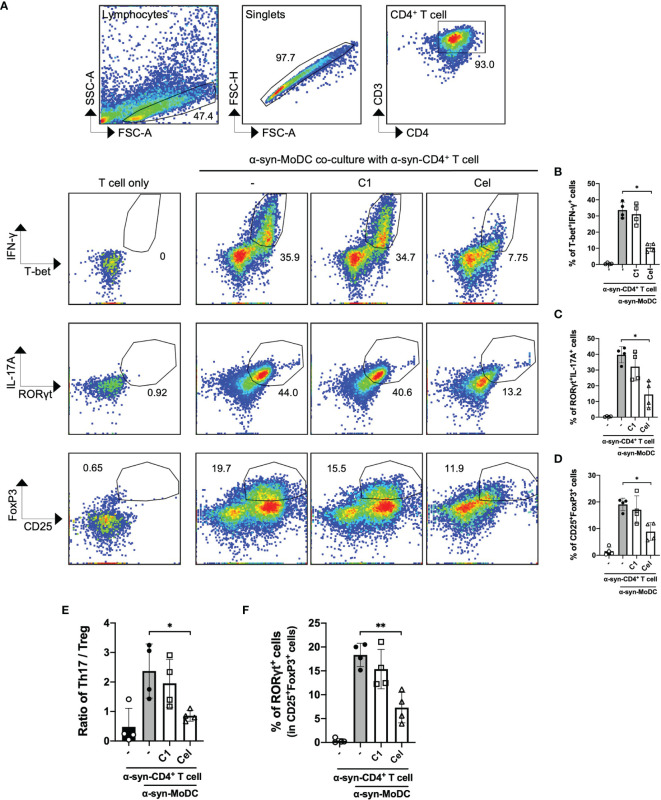
α-Syn-specific CD4^+^ T cell subsets stimulated by α-syn-pulsed MoDCs. α-syn specific CD4^+^ T cells (α-syn-CD4^+^ T cell) were co-cultured with MoDCs pre-treated with C1 (1 µM) or Celastrol (0.25 µM) for 1 h followed by α-syn treatment (α-syn-MoDC) and examined by flow cytometry. **(A)** Representative dot plots of 4 individual experiments showing frequencies of T-bet^+^IFN-γ^+^ (Th1), RORγt^+^IL-17A^+^ cells (Th17), and CD25^+^FoxP3^+^ (Treg) CD4^+^ T cells. α-syn-specific CD4^+^ T cells only served as the negative control. **(B–D)** Column graphs of flow cytometry results for the different subsets. **(E)** Ratio of Th17 to Treg in the co-culture conditions from 4 individual experiments. **(F)** Column graph showing percentage of RORγt expression in CD25^+^FoxP3^+^ (Treg) cells under different treatment conditions in 4 individual experiments. Column graph data represents mean ± SD from 4 individual experiments. Statistical significance was calculated by one-way ANOVA and Tukey’s multiple comparisons test, *P < 0.05, **P < 0.01.

Even though α-syn-treated MoDCs had minimal responses, we next sought to examine whether these cells are capable to induce T cell responses using a co-culture model. α-syn-specific CD4^+^ T cells were generated using a similar protocol (50, 56), then co-cultured (in a ratio of 5:1) with autologous α-syn-pulsed MoDCs with or without C1 or Celastrol pre-treatment. Flow cytometric analysis was performed to measure the abundance of T-bet^+^IFN-γ^+^ (Th1) on day 1, and RORγt^+^IL-17A^+^ (Th17) and CD25^+^FoxP3^+^ (Treg) cells on day 3, among CD3^+^CD4^+^ T cells after co-culture (**Supplementary Figure 4**). Data showed ~30% Th1 cells, ~40% Th17 cells, and ~20% Treg cells were stimulated by α-syn-pulsed MoDCs (**Figures 3A–D**). Of interest, within the Treg population stimulated by α-syn-pulsed MoDCs, ~20% of the cells co-expressed RORγt (**Figure 3F**). Celastrol pre-treatment of α-syn-pulsed MoDCs significantly reduced the frequencies of these T cell subsets, with 4-fold and 3.5-fold reduction in Th1 and Th17 cell frequencies, respectively (**Figures 3B, C**). It also improved the Th17/Treg ratio as there was a 2-fold decrease in Treg abundance (**Figures 3D, E**). In contrast, C1 pre-treatment resulted in a modest decrease in Th17 cells. We further found that Celastrol, but not C1, resulted in decreased FoxP3^−^RORγt^+^ and FoxP3^+^RORγt^+^ subpopulations while increased FoxP3^+^RORγt^−^ cells among CD3^+^CD4^+^CD25^+^ cells (**Supplementary Figure 4B**). Therefore, although α-syn treatment of MoDCs did not trigger the upregulation of antigen presentation molecules, α-syn-specific Th1, Th17, and Treg cells were still induced. Moreover, Celastrol appears to counteract this likely through a mechanism that does not involve the classic surface expressed MHC-II and co-stimulatory molecules.

### The Colocalization of α-Syn With Endo-Lysosomal Compartments is Modulated by Celastrol in MoDCs

**Figure 4:**
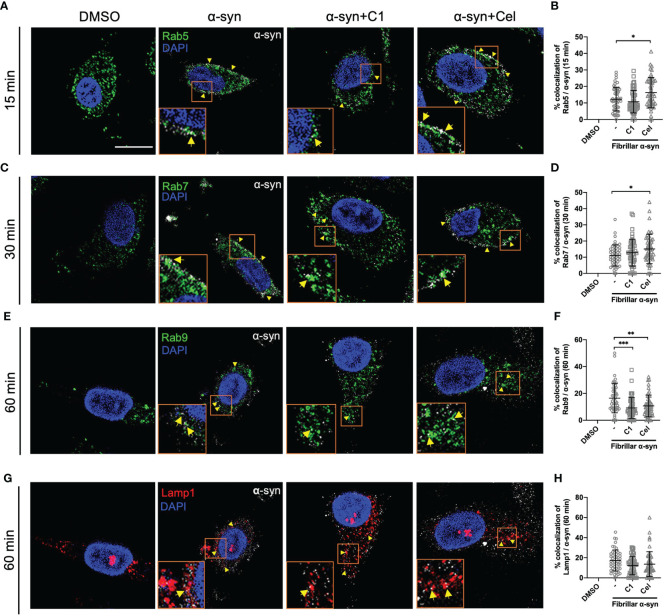
Intracellular Rab proteins colocalization with fibrillar α-syn in MoDCs. MoDCs were pre-treated with C1 (1 µM) or Celastrol (0.25 µM) for 1 h followed by treatment with fibrillar α-syn (1 µg/ml) for 15, 30 or 60 min. Rab5, Rab7, or Rab9 (green), Lamp1 (red) and α-syn (white) were immunostained for the corresponding timepoint with DAPI (blue) and observed under the confocal microscope. Representative images showing colocalization (yellow arrows) of Rab proteins with α-syn under different treatments and dot plots showing the percentage of colocalization. **(A, B)** Colocalization of Rab5 and α-syn at 15 min post α-syn treatment, **(C, D)** colocalization of Rab7 and α-syn at 30 min post α-syn treatment, **(E, F)** colocalization of Rab9 and α-syn at 60 min post α-syn treatment, and **(G, H)** colocalization of Lamp1 and α-syn at 60 min post α-syn treatment. Images are representative of 50 individual cells. Scale bar: 10 µm. Each dot in the dot plots represents data of a cell and the mean ± SD of 50 individual cells is indicated. Statistical significance was calculated by one-way ANOVA and Tukey’s multiple comparisons test, *P < 0.05, **P < 0.01, ***P < 0.001.

To understand how α-syn is trafficked in MoDCs leading to CD4^+^ T cell activation, and how Celastrol inhibited this, we next sought to examine the association of endo-lysosomal pathway proteins with α-syn in MoDCs. To this end, we tracked the colocalization of fibrillar α-syn with intracellular Rab5, Rab7, Rab9, Rab11, Rab14 proteins and lysosome marker Lamp1 at various timepoints using confocal microscopy. At 15 min, α-syn was colocalized with early endosomal marker Rab5, which implies that α-syn was likely uptaken through endocytosis by MoDCs (**Figure 4A**). At 30 min, trafficking to late endosomes occurred as α-syn was shown to colocalize with Rab7^+^ vesicles (**Figure 4C**). Another 30 min later, it appeared that α-syn containing vesicles were fused with lysosomes as they were colocalized with Lamp1 or Rab9, where the latter may mediate late endosome and lysosome fusion (**Figures 4E, G**). Interestingly, Celastrol increased the colocalization of α-syn with Rab5^+^ and Rab7^+^ vesicles (**Figures 4B, D**), but not with Rab9^+^ and Lamp1^+^ vesicles (**Figures 4F, H**). Besides, we also observed increased colocalization of α-syn with Rab14^+^ and Rab11^+^ vesicles under Celastrol treatment (**Supplementary Figure 5**), which are mainly found on early endosomes and recycling endosomes, respectively. However, this was unlikely a consequence of increased endosome formation as Celastrol had no significant upregulation on the number of Rab5^+^, Rab7^+^, Rab9^+^, Rab14^+^, Rab11^+^ and Lamp1^+^ vesicles in MoDCs (**Supplementary Figure 6**). On the other hand, C1 had no significant effect except decreased the number of Rab9 and α-syn colocalization (**Figure 4F**). These results suggest that α-syn could enter the endo-lysosomal pathway that may then be processed and generate α-syn peptides for antigen presentation.

### α-Syn is Found in Amphisomes in MoDCs

**Figure 5:**
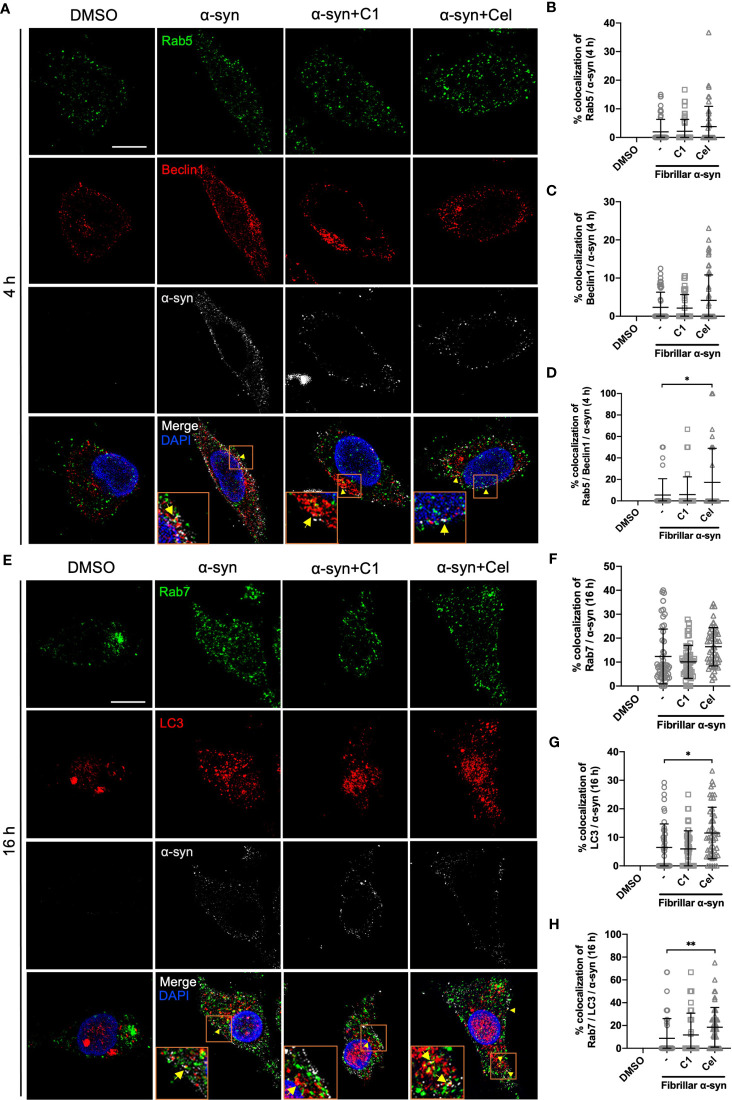
Colocalization of Rab proteins and autophagosome markers with fibrillar α-syn in MoDCs. MoDCs were pre-treated with C1 (1 µM) or Celastrol (0.25 µM) for 1 h followed by treatment with fibrillar α-syn (1 µg/ml) for 4 h or 16 h. Different Rab proteins (green) and autophagosome proteins (red) were immunostained for the corresponding timepoint along with α-syn (white) and DAPI (blue) and observed under the confocal microscope. **(A)** Representative images showing individual staining and merged images showing colocalization (yellow arrows) of Rab5 (green) and Beclin1 (red) with α-syn at 4 h post α-syn treatment. Scale bar: 10 µm. Dot plots showing the percentage of colocalization between **(B)** Rab5 and α-syn, **(C)** Beclin1 and α-syn and **(D)** Rab5, Beclin1 and α-syn. **(E)** Representative images showing individual staining and merged images showing colocalization (yellow arrows) of Rab7 (green) and LC3 (red) with α-syn at 16 h post α-syn treatment. Scale bar: 10 µm. Dot plots showing the percentage of colocalization between **(F)** Rab7 and α-syn, **(G)** LC3 and α-syn and **(H)** Rab7, LC3 and α-syn. Images are representative of 50 individual cells. Each dot in the dot plots represents data of a cell and the mean ± SD of 50 individual cells is indicated. Statistical significance was calculated by one-way ANOVA and Tukey’s multiple comparisons test, *P < 0.05, **P < 0.01.

**Figure 6:**
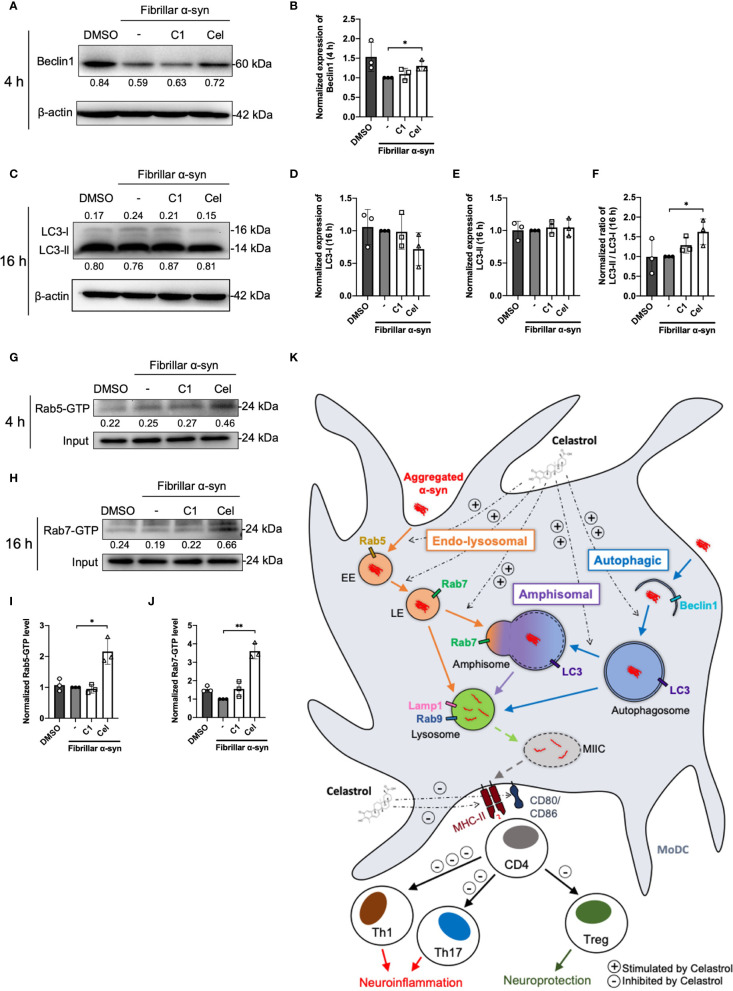
Autophagosome markers and Rab-GTP protein expression level in α-syn treated MoDCs. **(A)** The expression of Beclin1 in drug pre-treated MoDCs was determined by Western Blot at 4 h post α-syn treatment. Relative expressions of Beclin1 to β-actin were quantified by ImageJ and indicated on the blots, which were further normalized to α-syn only treatment shown in **(B)**. Column graph data represents mean ± SD from 3 individual experiments. **(C)** The expressions of LC3-I and LC3-II in drug pre-treated MoDCs were determined by Western Blot at 16 h post α-syn treatment. Relative expressions of LC3-I or LC3-II to β-actin were quantified using ImageJ and indicated on the blots, which were further normalized to α-syn only treatment shown in **(D, E)**. **(F)** The ratio of LC3-II to LC3-I in each treatment was calculated and further normalized to α-syn only treatment. Column graph data represents mean ± SD from 3 individual experiments. **(G)** Active Rab5-GTP **(H)** and Rab7-GTP were immunoprecipitated and their relative expressions towards whole cell lysate input control were quantified by ImageJ and indicated on blots, which were further normalized to α-syn only treatment shown in **(I, J)**. Column graph data represents mean ± SD from 3 individual experiments. Statistical significance was calculated by one-way ANOVA and Tukey’s multiple comparisons test, *P < 0.05, **P < 0.01. **(K)** Schematic diagram of α-syn aggregates trafficking in MoDCs underlying antigen presentation and CD4^+^ T cell activation. Following uptake, α-syn interacts with components of three antigen trafficking and processing pathways: 1) Endo-lysosomal pathway (orange) beginning from Rab5^+^ early endosome (EE) to Rab7^+^ late endosome (LE) and to Rab9^+^/Lamp1^+^ lysosome degradation; 2) autophagic pathway (blue) starting from autophagosome formation (Beclin1^+^) enclosing aggregated α-syn, matured with LC3 and then to Rab9^+^/Lamp1^+^ lysosome degradation; 3) amphisomal pathway (purple) involves the fusion of Rab7^+^ LE from the endo-lysosomal pathway with LC3^+^ autophagosome from the autophagic pathway, which may also be regulated by the formation of Rab5^+^/Beclin1^+^ vesicles, and consequently fuse with Rab9^+^/Lamp1^+^ lysosomes. These three pathways could possibly allow the processing of the encapsulated α-syn by lysosomes and provide α-syn antigen peptides at different extent for MHC-II presentation to CD4^+^ T cells and trigger differentiation into Th1, Th17 and Treg subsets that may contribute to either neuroinflammation or neuroprotection. Celastrol promotes the colocalization of α-syn with Rab5^+^ EE and Rab7^+^ LE in the endo-lysosomal pathway, with autophagosome in the autophagic pathway, and the formation of amphisome containing α-syn in the amphisomal pathway. The increased α-syn interaction with components from degradation pathways likely favored the processing of α-syn and reduced α-syn peptides presentation to CD4^+^ T cell and decreased the frequencies of Th1, Th17 and Treg subsets.

The endo-lysosomal pathway can converge with the autophagic pathway through fusion of autophagosomes with late endosomes to form amphisomes, which the engulfed proteins can be directed for degradation when fused with lysosomes (57, 58). Rab5 can be involved in autophagosome formation and, conversely, Beclin1 is also associated with Rab5-mediated endosomal trafficking (59, 60). Thus, we next tested whether fibrillar α-syn is also trafficked to autophagic and amphisomal components in MoDCs. MoDCs were treated with fibrillar α-syn for 4 h or 16 h and assessed for colocalization of autophagy-related Beclin1 and LC3 with α-syn, and their association with Rab5 and Rab7 proteins, respectively. Expectedly, Beclin1 or LC3 were found colocalized with α-syn (**Figures 5A, C, E, G**). Rab5 or Rab7 were also found colocalized in these puncta, indicating the formation of amphisomes (**Figures 5A, D, E, H** and **Supplementary Figure 7**). These data suggest that apart from the endo-lysosomal pathway, autophagic pathway is also associated with the trafficking of α-syn in MoDCs, where the formation of LC3^+^Rab7^+^ amphisomes occurred, indicating the convergence of the two pathways. Moreover, Celastrol, but not C1, markedly increased the number of Rab5/Beclin1/α-syn and Rab7/LC3/α-syn puncta (**Figures 5D, H**), and also the autophagic Beclin1^+^ and LC3^+^ puncta (**Supplementary Figures 8B, E**). Also, Rab7/LC3 amphisomes were increased by 2.2-fold under Celastrol treatment, suggesting that Celastrol can induce both the autophagic and amphisomal vesicles interaction with α-syn (**Supplementary Figure 8F**). To confirm the confocal microscopy data, western blot analysis was performed on MoDCs at 4 and 16 h post α-syn treatment. Beclin1 protein level was found to be downregulated following α-syn treatment compared to control but appears to be rescued by Celastrol treatment (**Figures 6A, B**). Similarly, while the protein level of LC3-I or LC3-II had no significant change after α-syn treatment, Celastrol increased the LC3-II : LC3-I conversion ratio (**Figures 6E, F**). Furthermore, we tested the expression of p62 in α-syn treated MoDCs, where its decreased expression indicates autophagy flux. We found that α-syn treatment upregulated p62 expression which was counteracted by Celastrol (**Supplementary Figures 9C, D**). Moreover, an increased p62 colocalization with α-syn in Celastrol pre-treated MoDCs was observed (**Supplementary Figures 9A, B**). These data largely supported the notion that fibrillar α-syn can be encapsulated in autophagic components which likely allowed the processing of α-syn through autophagy. However, this happens to a lesser extent in MoDCs but can be alleviated by Celastrol. Lastly, the level of active GTP forms of Rab5 and Rab7 were upregulated by Celastrol pre-treatment (**Figures 6G–J**), in corroboration with the above data. Together, Celastrol not only induced autophagy by upregulating Beclin1 expression, reducing p62 expression and increasing LC3 conversion, but it can also trigger the amphisomal pathway with increased functional Rab5 and Rab7 proteins to interact with autophagic components for trafficking of α-syn towards the lysosomal-mediated processing.

## Discussion

Neuroinflammation aroused by peripheral immune cells in response to abnormal α-syn has been highlighted in the progression of PD (30, 61). In particular, the involvement of DCs in mediating α-syn-specific T cells that causes detrimental effects to dopaminergic neurons has been reported with elevated pro-inflammatory Th1 and Th17 subsets in PD patients (12, 16, 62). However, the underlying mechanism that triggers these DC-T responses remains poorly understood. Herein, we demonstrated that fibrillar α-syn were trafficked to both endo-lysosomal and autophagic pathways in MoDCs, suggesting the use of these pathways for lysosomal processing of α-syn. However, these processes were altered by the fibrillar α-syn protein aggregate itself such as the suppression of autophagic pathways, but the endo-lysosomal pathway still resulted in α-syn peptides that may be used for antigen presentation for the activation of Th1 and Th17 cells. In parallel, we also tested the effectiveness of two TCM compounds (C1 and Celastrol) that may modulate this mechanism. While C1 and Celastrol can promote autophagy (45–47), and the latter can suppress inflammatory response (48), nevertheless, we found that only Celastrol promoted the colocalization of fibrillar α-syn with endo-lysosomal, amphisomal and autophagic markers in MoDCs. The likelihood of these events lies in Celastrol being able to promote the complete degradation of α-syn, which leads to decreased antigenic peptides available for antigen presentation, and thereby reduced the T cell responses. Overall, this study provided insights into an antigen trafficking-related immune mechanism in regulating pro-inflammatory α-syn-specific T cells for potential therapeutic intervention against PD.

While most studies of T cell polarization in the brain focused on the roles of microglia and astrocytes (63, 64), here, we demonstrated the ability of peripheral MoDCs in triggering pro-inflammatory α-syn-specific CD4^+^ T cell differentiation in the co-culture experiments. A significant proportion of Th1 and Th17 cells was found compared to Treg cells (**Figure 3**). A shift of pro-inflammatory Th1 and Th17 cell subsets in PD patients had also been reported (16, 65), which likely contributed to the development of neuroinflammation through secreting pro-inflammatory cytokines IFN-γ and IL-17A that are elevated in the serum of PD patients (17). These cytokines could lead to neuronal cell death (24, 66), activate neurotoxic M1-like microglia and astrocytes (67), promote macrophages and B cells infiltration into the brain (68), and also inhibit Treg function in neuronal repair and suppression of pro-inflammatory T cells cytotoxicity towards dopaminergic neurons (69–71). Therefore, the balance of the pro-inflammatory and regulatory T cell subsets is important to prevent the progression of PD, where intervention on DC response to α-syn may shed light.

To date, whether fibrillar α-syn could induce APC activation remains controversial. Some studies reported an upregulation of MHC and co-stimulatory molecules in α-syn treated DCs, while others found no differences (or even a decrease) in α-syn processing and presentation in patient-derived DCs compared to healthy control (31, 32, 72, 73). In our results, fibrillar α-syn did not upregulate MHC-II and co-stimulatory molecules expression on MoDCs or cytokine production (**Figures 1**, **2**). Indeed, oligomeric β-amyloid in Alzheimer’s Disease (AD) was also reported to have no effect on MHC-II expression on brain-derived or bone marrow-derived DCs (74), or even a reduction in the abundance of MHC-II^hi^ DCs in mice (75). Taken together with our data, brain self-proteins appear to have a minimal upregulation effect on DC antigen presenting capacity, which may allow the escape of immune surveillance of α-syn and β-amyloid associated with T cells leading to detrimental protein aggregation (74, 76). In our experiments, however, α-syn-pulsed MoDCs could still trigger α-syn-specific T cell responses that may drive neuroinflammation in PD. Whether they have any effects on α-syn-expressing neurons remains to be investigated. Moreover, the presentation to and induction of the α-syn-specific T cells could be attributed to mechanisms besides DC-T immunological synapse formation. One possibility could be related to exosomes derived from APC. The role of exosomes in initiating inflammatory T cells has been reported with α-syn-activated microglia (77), and exosomes derived from MoDCs express surface MHC-I/II and co-stimulatory molecules that can activate antigen-specific CD4^+^ T cells (78–80). One report demonstrated DC-derived exosomes could stimulate antigen-specific CD4^+^ T cells in the presence of MHC-II-deficient DCs in mice (81), which may be one possible scenario in our experimental co-culture of α-syn-pulsed MoDCs and the α-syn-specific CD4^+^ T cell responses. Although we did not address the involvement of exosomes directly, we did observe the interaction between the recycling endosomal marker Rab11 and α-syn, where Rab11 has been reported to facilitate exosome formation (82). Further investigation on exosomes derived from α-syn-pulsed MoDCs may explain how they trigger α-syn-specific T cell activation and differentiation during PD.

Endo-lysosomal or autophagic pathway for degradation of antigen and loading onto MHC-II are important in regulating efficient antigen presentation to CD4^+^ T cells (41, 83, 84). It remains poorly understood how α-syn peptides are generated for MHC-II antigen presentation, especially in DCs. Our data showed that both endo-lysosomal and autophagic components are associated with fibrillar α-syn in MoDCs (**Figures 4**, **5**), indicating the intracellular trafficking of α-syn into these pathways. α-syn were initially found enclosed in early Rab5^+^ endosomes, which were then trafficked to late Rab7^+^ endosomes (**Figures 4A–D**), in which Rab5 to Rab7 conversion was reported to be important for effective antigen degradation and presentation (85). Lastly, fusion of α-syn containing vesicles with Lamp1^+^ lysosomes occurred which could be an indication of lysosomal degradation of α-syn (**Figures 4G, H**), and is regulated by Rab9 (**Figures 4E–H**). This likely generated α-syn peptides that may be transported to the MHC-II compartment (MIIC) where loading of α-syn peptides onto the MHC-II for presentation to CD4^+^ T cells takes place (86–88). On the other hand, trafficking of fibrillar α-syn into autophagic pathway was also observed. α-Syn were found colocalized with Beclin1 and later with LC3 in MoDCs (**Figure 5**). Moreover, these two pathways can also converge to initiate the formation of amphisome, which is the fusion of early/late endosome with autophagosome where the content will be degraded by lysosomes (89, 90). Indeed, besides autophagy, this alternative pathway has been reported for cargo degradation and MHC-II presentation in processing bacterial and tumor antigens in B cells and DCs (87, 88, 91, 92). We observed the presence of Rab5/Beclin1/α-syn and Rab7/LC3/α-syn puncta which indicates that fibrillar α-syn can also be found in amphisomes in MoDCs (**Figure 5** and **Supplementary Figure 7**). In short, our data provide evidence that intracellular trafficking of α-syn can be associated with three pathways in MoDCs: endo-lysosomal pathway, autophagic pathway and amphisomal pathway for lysosomal-mediated degradation. However, the efficiency of these pathways in degrading α-syn appeared to be modulated by α-syn. Previous studies reported that limited antigen degradation capacity favors the generation of MHC-II peptides which can be counteracted by increased autophagy (41, 93, 94). The pathway and the association of the fibrillar α-syn processing that is the most important for MoDCs in antigen presentation to stimulate pro-inflammatory or regulatory α-syn-specific CD4^+^ T cell responses in PD remains to be investigated.

In the attempt to uncover potential TCM against PD and the effect on the underlying immune mechanisms, we investigated whether *Curcumin* analog C1 or TWHF-derived Celastrol can modulate α-syn interaction with Rab and autophagy-related proteins that may underly the activation of α-syn-specific T cells by MoDCs. C1 is an activator of TFEB, which is an important inducer of autophagy (45); but it failed to promote the interaction of endosomes, amphisomes and autophagosomes with fibrillar α-syn in MoDCs (**Figures 4**, **5**), and only resulted in a slight reduction of Th1 and Th17 frequencies (**Figure 3**). In contrast, Celastrol demonstrated immune-modulating effects throughout our experiments. First, it limited T cell activation by reducing cytokine gene transcription capacity of MoDCs and decreasing MHC-II and co-stimulatory molecules expression (**Figures 1**, **2**). Second, it enhanced fibrillar α-syn colocalization with Rab5 early endosomes and Rab7 late endosomes (**Figure 4**). Third, it promoted autophagic pathways with increased Beclin1 protein level, and enhanced LC3-I to LC3-II conversion which indicate autophagosome maturation and possibly promoted autophagosome degradation indicated from the decreased p62 expression. It also increased colocalization between α-syn and p62^+^ vesicles or LC3^+^ autophagosomes, enhanced Rab protein activities and their colocalizations with autophagosomes and α-syn for amphisomes (**Figures 5**, **6** and **Supplementary Figures 7–9**). Taken together, these events possibly aided the trafficking of α-syn for degradation by lysosomes and likely resulted in excessive α-syn processing (or complete degradation) that led to the unavailability of antigenic peptides to be loaded onto MHC-II (95). Thereby, brought about a significant reduction of pro-inflammatory T cell subsets and Th17/Treg ratio (**Figure 3**). Therefore, apart from being an autophagy inducer for the degradation of aggregated α-syn, Celastrol showed its anti-inflammatory function in regulating MoDC-T cell responses in PD. Besides PD, studies have shown the anti-inflammatory role of Celastrol in other autoimmune diseases. For instance, it can regulate NF-κB and provides neuroprotection by suppressing pro-inflammatory cytokines TNF-α and IL-1β in AD brain, downregulate IL-6 and IL-17 expression in rheumatoid arthritis mice model, or suppress microgliosis and inflammatory cytokine production in the optic nerve of experimental autoimmune encephalomyelitis model (48, 96–98). However, whether Celastrol can dampen autoreactive inflammatory DC-T cell inflammatory responses in these diseases remain to be investigated. Altogether, Celastrol can downmodulate α-syn-specific T cell responses due to the enhancement of autophagic and amphisomal pathways.

The specific targets for Celastrol to mediate these pathways remain elusive. Thus, we used the SwissTargetPrediction tool and the STRING database to identify putative targets (**Supplementary Figures 11, 12**). Some possibilities for Celastrol include proteins involved in arachidonic acid (AA) inflammatory pathways (e.g., PLA2G4A), where AA is elevated in PD mice model and also associated with Rab5 endocytosis and antigen presentation (99–102). The function of PLA2G4A is to catalyze the release of AA and it is found expressed in DCs, where induced expression can lead to inflammatory T cell responses (103, 104). Others also found its role in autophagy flux and neuronal loss post brain trauma (105). Another potential target, PTPN11, is involved in the MAPK-signaling pathway, and this protein was reported to be crucial for the induction of DC-mediated Th17 activities in anti-fungal responses (106). Androgen receptor (AR) is a known target of Celastrol to induce autophagy in cancers (107), but its role in PD is not understood. However, AR can be destabilized by Celastrol and result in the regulation of peripheral T cell proliferation and Th1 differentiation to inhibit autoimmune diseases (108, 109). On the other hand, the targets of Celastrol are not associated with C1, where the potential protein targets appear to be more diverse in functions (**Supplementary Figure 12**). Only cathepsin (CTS) is involved in α-syn degradation (110) (**Supplementary Figures 10, 12**). Altogether, the putative targets of Celastrol are more specific in regulating inflammatory T cell responses, where an explanation of its effect in our DC-T cell co-culture experiments lies within. Overall, this study provided additional understanding of Celastrol to be used as a treatment for PD in the reduction of DC-mediated pro-inflammatory T cells against α-syn.

## Data Availability Statement

The raw data supporting the conclusions of this article will be made available by the authors, without undue reservation.

## Ethics Statement

The studies involving human participants were reviewed and approved by the Hong Kong Baptist University Research Ethics Committee (#REC/19-20/0110). Written informed consent for participation was not required for this study in accordance with the national legislation and the institutional requirements.

## Author Contributions

LN and AC designed and performed the experiments, analyzed the data, and wrote the manuscript. XW conducted the BV2 experiments. CY, CS and ML provided reagents and critical comments. CY, ML and AC supervised the study. All authors listed have made a substantial, direct, and intellectual contribution to the work and approved it for publication.

## Funding

This study was supported by the Health and Medical Research Fund (HMRF) (18170032), the Interdisciplinary Research Matching Scheme (RC-IRCs-1718-03), the Faculty Research Grant (FRG2/17–18/066), the Faculty Start-up Fund (SCI-17-18-01), the Tier 2 Start-up Grant (RC-SGT2/18–19/SCI/007), and the Research Council Start-up Grant of Hong Kong Baptist University (to AC). This study was also partly supported by HMRF17182541, HMRF17182551, GRF/HKBU12100618 from the Hong Kong Government, the National Natural Science Foundation of China (81703487, and 81773926), and the Shenzhen Science and Technology Innovation Commission (JCYJ20180302174028790, JCYJ20180507184656626) (to ML).

## Conflict of Interest

The authors declare that the research was conducted in the absence of any commercial or financial relationships that could be construed as a potential conflict of interest.

## Publisher’s Note

All claims expressed in this article are solely those of the authors and do not necessarily represent those of their affiliated organizations, or those of the publisher, the editors and the reviewers. Any product that may be evaluated in this article, or claim that may be made by its manufacturer, is not guaranteed or endorsed by the publisher.

## References

[1] Hirsch L Jette N Frolkis A Steeves T Pringsheim T. The Incidence of Parkinson’s Disease: A Systematic Review and Meta-Analysis. Neuroepidemiology (2016) 46(4):292–300. doi: 10.1159/000445751 27105081

[2] Michel PP Hirsch EC Hunot S. Understanding Dopaminergic Cell Death Pathways in Parkinson Disease. Neuron (2016) 90(4):675–91. doi: 10.1016/j.neuron.2016.03.038 27196972

[3] Kahle PJ Neumann M Ozmen L Haass C. Physiology and Pathophysiology of Alpha-Synuclein. Cell Culture and Transgenic Animal Models Based on a Parkinson’s Disease-Associated Protein. Ann N Y Acad Sci (2000) 920:33–41. doi: 10.1111/j.1749-6632.2000.tb06902.x 11193173

[4] Burré J Sharma M Tsetsenis T Buchman V Etherton MR Südhof TC. Alpha-Synuclein Promotes SNARE-Complex Assembly In Vivo and *In Vitro* . Science (2010) 329(5999):1663–7. doi: 10.1126/science.1195227 PMC3235365 20798282

[5] Ball N Teo WP Chandra S Chapman J. Parkinson’s Disease and the Environment. Front Neurol (2019) 10:218. doi: 10.3389/fneur.2019.00218 30941085 PMC6433887

[6] Maries E Dass B Collier TJ Kordower JH Steece-Collier K. The Role of Alpha-Synuclein in Parkinson’s Disease: Insights From Animal Models. Nat Rev Neurosci (2003) 4(9):727–38. doi: 10.1038/nrn1199 12951565

[7] Petrucelli L O’Farrell C Lockhart PJ Baptista M Kehoe K Vink L. Parkin Protects Against the Toxicity Associated With Mutant Alpha-Synuclein: Proteasome Dysfunction Selectively Affects Catecholaminergic Neurons. Neuron (2002) 36(6):1007–19. doi: 10.1016/s0896-6273(02)01125-x 12495618

[8] Bousset L Pieri L Ruiz-Arlandis G Gath J Jensen PH Habenstein B. Structural and Functional Characterization of Two Alpha-Synuclein Strains. Nat Commun (2013) 4:2575. doi: 10.1038/ncomms3575 24108358 PMC3826637

[9] Yacoubian TA Standaert DG. Reaping What You Sow: Cross-Seeding Between Aggregation-Prone Proteins in Neurodegeneration. Mov Disord (2014) 29(3):306. doi: 10.1002/mds.25766 24395732

[10] Cebrián C Loike JD Sulzer D. Neuroinflammation in Parkinson’s Disease Animal Models: A Cell Stress Response or a Step in Neurodegeneration? Curr Top Behav Neurosci (2015) 22:237–70. doi: 10.1007/7854_2014_356 25293443

[11] Li Q Barres BA. Microglia and Macrophages in Brain Homeostasis and Disease. Nat Rev Immunol (2018) 18(4):225–42. doi: 10.1038/nri.2017.125 29151590

[12] Brochard V Combadière B Prigent A Laouar Y Perrin A Beray-Berthat V. Infiltration of CD4+ Lymphocytes Into the Brain Contributes to Neurodegeneration in a Mouse Model of Parkinson Disease. J Clin Invest (2009) 119(1):182–92. doi: 10.1172/jci36470 PMC2613467 19104149

[13] Hatterer E Touret M Belin M-F Honnorat J Nataf S. Cerebrospinal Fluid Dendritic Cells Infiltrate the Brain Parenchyma and Target the Cervical Lymph Nodes Under Neuroinflammatory Conditions. PloS One (2008) 3(10):e3321. doi: 10.1371/journal.pone.0003321 18830405 PMC2552991

[14] Louveau A Harris TH Kipnis J. Revisiting the Mechanisms of CNS Immune Privilege. Trends Immunol (2015) 36(10):569–77. doi: 10.1016/j.it.2015.08.006 PMC4593064 26431936

[15] Stevens CH Rowe D Morel-Kopp MC Orr C Russell T Ranola M. Reduced T Helper and B Lymphocytes in Parkinson’s Disease. J Neuroimmunol (2012) 252(1-2):95–9. doi: 10.1016/j.jneuroim.2012.07.015 22910543

[16] Kustrimovic N Comi C Magistrelli L Rasini E Legnaro M Bombelli R. Parkinson’s Disease Patients Have a Complex Phenotypic and Functional Th1 Bias: Cross-Sectional Studies of CD4+ Th1/Th2/T17 and Treg in Drug-Naïve and Drug-Treated Patients. J Neuroinflamm (2018) 15(1):205. doi: 10.1186/s12974-018-1248-8 PMC6044047 30001736

[17] Yang F Li B Li L Zhang H. The Clinical Significance of the Imbalance of Th 17 and Treg Cells and Their Related Cytokines in Peripheral Blood of Parkinson’s Disease Patients. Int J Clin Exp Med (2016) 9(9):17946–51.

[18] Huang Y Liu Z Cao B-B Qiu Y-H Peng Y-P. Treg Cells Attenuate Neuroinflammation and Protect Neurons in a Mouse Model of Parkinson’s Disease. J Neuroimmune Pharmacol (2020) 15(2):224–37. doi: 10.1007/s11481-019-09888-5 31802419

[19] Revu S Wu J Henkel M Rittenhouse N Menk A Delgoffe GM. IL-23 and IL-1β Drive Human Th17 Cell Differentiation and Metabolic Reprogramming in Absence of CD28 Costimulation. Cell Rep (2018) 22(10):2642–53. doi: 10.1016/j.celrep.2018.02.044 PMC5884137 29514093

[20] Storelli E Cassina N Rasini E Marino F Cosentino M. Do Th17 Lymphocytes and IL-17 Contribute to Parkinson’s Disease? A Systematic Review of Available Evidence. Front Neurol (2019) 10:13(13). doi: 10.3389/fneur.2019.00013 30733703 PMC6353825

[21] Zúñiga LA Jain R Haines C Cua DJ. Th17 Cell Development: From the Cradle to the Grave. Immunol Rev (2013) 252(1):78–88. doi: 10.1111/imr.12036 23405896

[22] Lindestam Arlehamn CS Dhanwani R Pham J Kuan R Frazier A Rezende Dutra J. α-Synuclein-Specific T Cell Reactivity Is Associated With Preclinical and Early Parkinson’s Disease. Nat Commun (2020) 11(1):1875. doi: 10.1038/s41467-020-15626-w 32313102 PMC7171193

[23] Sulzer D Alcalay RN Garretti F Cote L Kanter E Agin-Liebes J. T Cells From Patients With Parkinson’s Disease Recognize α-Synuclein Peptides. Nature (2017) 546(7660):656–61. doi: 10.1038/nature22815 PMC5626019 28636593

[24] Sommer A Marxreiter F Krach F Fadler T Grosch J Maroni M. Th17 Lymphocytes Induce Neuronal Cell Death in a Human iPSC-Based Model of Parkinson’s Disease. Cell Stem Cell (2018) 23(1):123–31.e126. doi: 10.1016/j.stem.2018.06.015 29979986

[25] Kapsenberg ML. Dendritic-Cell Control of Pathogen-Driven T-Cell Polarization. Nat Rev Immunol (2003) 3(12):984–93. doi: 10.1038/nri1246 14647480

[26] Sprent J. Antigen-Presenting Cells. Professionals and Amateurs. Curr Biol (1995) 5(10):1095–7. doi: 10.1016/s0960-9822(95)00219-3 8548275

[27] Benkler M Agmon-Levin N Shoenfeld Y. Parkinson’s Disease, Autoimmunity, and Olfaction. Int J Neurosci (2009) 119(12):2133–43. doi: 10.3109/00207450903178786 19916845

[28] Ludewig P Gallizioli M Urra X Behr S Brait VH Gelderblom M. Dendritic Cells in Brain Diseases. Biochim Biophys Acta (2016) 1862(3):352–67. doi: 10.1016/j.bbadis.2015.11.003 26569432

[29] Ciaramella A Salani F Bizzoni F Pontieri FE Stefani A Pierantozzi M. Blood Dendritic Cell Frequency Declines in Idiopathic Parkinson’s Disease and is Associated With Motor Symptom Severity. PloS One (2013) 8(6):e65352. doi: 10.1371/journal.pone.0065352 23776473 PMC3679103

[30] Harms AS Thome AD Yan Z Schonhoff AM Williams GP Li X. Peripheral Monocyte Entry is Required for Alpha-Synuclein Induced Inflammation and Neurodegeneration in a Model of Parkinson Disease. Exp Neurol (2018) 300:179–87. doi: 10.1016/j.expneurol.2017.11.010 PMC5759972 29155051

[31] Alam MM Yang D Trivett AL Li X-Q Oppenheim JJ. Alpha-Synuclein (αs) Acts as an Alarmin to Promote Dendritic Cell Activation and Proinflammatory Immune Response. J Immunol (2019) 202(1 Supplement):68.17–7.

[32] Álvarez-Luquín DD Arce-Sillas A Leyva-Hernández J Sevilla-Reyes E Boll MC Montes-Moratilla E. Regulatory Impairment in Untreated Parkinson’s Disease Is Not Restricted to Tregs: Other Regulatory Populations Are Also Involved. J Neuroinflamm (2019) 16(1):212. doi: 10.1186/s12974-019-1606-1 PMC6849192 31711508

[33] Burgdorf S Kurts C. Endocytosis Mechanisms and the Cell Biology of Antigen Presentation. Curr Opin Immunol (2008) 20(1):89–95. doi: 10.1016/j.coi.2007.12.002 18249105

[34] Iwasaki A Medzhitov R. Control of Adaptive Immunity by the Innate Immune System. Nat Immunol (2015) 16(4):343–53. doi: 10.1038/ni.3123 PMC4507498 25789684

[35] Prashar A Schnettger L Bernard EM Gutierrez MG. Rab GTPases in Immunity and Inflammation. Front Cell Infect Microbiol (2017) 7:435. doi: 10.3389/fcimb.2017.00435 29034219 PMC5627064

[36] Brundin P Dave KD Kordower JH. Therapeutic Approaches to Target Alpha-Synuclein Pathology. Exp Neurol (2017) 298(Pt B):225–35. doi: 10.1016/j.expneurol.2017.10.003 PMC6541231 28987463

[37] Decressac M Mattsson B Weikop P Lundblad M Jakobsson J Björklund A. TFEB-Mediated Autophagy Rescues Midbrain Dopamine Neurons From α-Synuclein Toxicity. Proc Natl Acad Sci USA (2013) 110(19):E1817–26. doi: 10.1073/pnas.1305623110 PMC3651458 23610405

[38] Bonam SR Tranchant C Muller S. Autophagy-Lysosomal Pathway as Potential Therapeutic Target in Parkinson’s Disease. Cells (2021) 10(12):3547. doi: 10.3390/cells10123547 34944054 PMC8700067

[39] Kubo M Nagashima R Kurihara M Kawakami F Maekawa T Eshima K. Leucine-Rich Repeat Kinase 2 Controls Inflammatory Cytokines Production Through NF-κb Phosphorylation and Antigen Presentation in Bone Marrow-Derived Dendritic Cells. Int J Mol Sci (2020) 21(5):1890. doi: 10.3390/ijms21051890 PMC7084871 32164260

[40] Freeman D Cedillos R Choyke S Lukic Z McGuire K Marvin S. Alpha-Synuclein Induces Lysosomal Rupture and Cathepsin Dependent Reactive Oxygen Species Following Endocytosis. PloS One (2013) 8(4):e62143. doi: 10.1371/journal.pone.0062143 23634225 PMC3636263

[41] Manoury B. Proteases: Essential Actors in Processing Antigens and Intracellular Toll-Like Receptors. Front Immunol (2013) 4:299. doi: 10.3389/fimmu.2013.00299 24065969 PMC3781364

[42] Junutula JR De Maziére AM Peden AA Ervin KE Advani RJ van Dijk SM. Rab14 Is Involved in Membrane Trafficking Between the Golgi Complex and Endosomes. Mol Biol Cell (2004) 15(5):2218–29. doi: 10.1091/mbc.e03-10-0777 PMC404017 15004230

[43] Barth S Glick D Macleod KF. Autophagy: Assays and Artifacts. J Pathol (2010) 221(2):117–24. doi: 10.1002/path.2694 PMC2989884 20225337

[44] Szatmári Z Sass M. The Autophagic Roles of Rab Small GTPases and Their Upstream Regulators: A Review. Autophagy (2014) 10(7):1154–66. doi: 10.4161/auto.29395 PMC4203544 24915298

[45] Song JX Sun YR Peluso I Zeng Y Yu X Lu JH. A Novel Curcumin Analog Binds to and Activates TFEB In Vitro and In Vivo Independent of MTOR Inhibition. Autophagy (2016) 12(8):1372–89. doi: 10.1080/15548627.2016.1179404 PMC4968239 27172265

[46] Liu X Zhao P Wang X Wang L Zhu Y Song Y. Celastrol Mediates Autophagy and Apoptosis via the ROS/JNK and Akt/mTOR Signaling Pathways in Glioma Cells. J Exp Clin Cancer Res (2019) 38(1):184. doi: 10.1186/s13046-019-1173-4 31053160 PMC6500040

[47] Shi J Li J Xu Z Chen L Luo R Zhang C. Celastrol: A Review of Useful Strategies Overcoming its Limitation in Anticancer Application. Front Pharmacol (2020) 11:558741. doi: 10.3389/fphar.2020.558741 33364939 PMC7751759

[48] Allison AC Cacabelos R Lombardi VR Alvarez XA Vigo C. Celastrol, a Potent Antioxidant and Anti-Inflammatory Drug, as a Possible Treatment for Alzheimer’s Disease. Prog Neuropsychopharmacol Biol Psychiatry (2001) 25(7):1341–57. doi: 10.1016/s0278-5846(01)00192-0 11513350

[49] Liu J Lee J Salazar Hernandez MA Mazitschek R Ozcan U. Treatment of Obesity With Celastrol. Cell (2015) 161(5):999–1011. doi: 10.1016/j.cell.2015.05.011 26000480 PMC4768733

[50] Mo Y Cheung AKL Liu Y Liu L Chen Z. Imaging and Analysis on the Interaction Between Human Antigen-Pulsed Vδ2 T Cells and Antigen-Specific CD4 T Cells. STAR Protoc (2021) 2(2):100453. doi: 10.1016/j.xpro.2021.100453 33937873 PMC8076705

[51] Schneider CA Rasband WS Eliceiri KW. NIH Image to ImageJ: 25 Years of Image Analysis. Nat Methods (2012) 9(7):671–5. doi: 10.1038/nmeth.2089 PMC5554542 22930834

[52] Szklarczyk D Gable AL Nastou KC Lyon D Kirsch R Pyysalo S. The STRING Database in 2021: Customizable Protein-Protein Networks, and Functional Characterization of User-Uploaded Gene/Measurement Sets. Nucleic Acids Res (2021) 49(D1):D605–12. doi: 10.1093/nar/gkaa1074 33237311 PMC7779004

[53] Lu YC Yeh WC Ohashi PS. LPS/TLR4 Signal Transduction Pathway. Cytokine (2008) 42(2):145–51. doi: 10.1016/j.cyto.2008.01.006 18304834

[54] Mbongue JC Nieves HA Torrez TW Langridge WH. The Role of Dendritic Cell Maturation in the Induction of Insulin-Dependent Diabetes Mellitus. Front Immunol (2017) 8:327. doi: 10.3389/fimmu.2017.00327 28396662 PMC5366789

[55] Trevejo JM Marino MW Philpott N Josien R Richards EC Elkon KB. TNF-Alpha -Dependent Maturation of Local Dendritic Cells Is Critical for Activating the Adaptive Immune Response to Virus Infection. Proc Natl Acad Sci U.S.A. (2001) 98(21):12162–7. doi: 10.1073/pnas.211423598 PMC59785 11593031

[56] Mo Y Cheung AKL Liu Y Liu L Chen Z. Delta42PD1-TLR4 Augments Gammadelta-T Cell Activation of the Transitional Memory Subset of CD4(+) T Cells. iScience (2020) 23(10):101620. doi: 10.1016/j.isci.2020.101620 33089108 PMC7567942

[57] Baixauli F López-Otín C Mittelbrunn M. Exosomes and Autophagy: Coordinated Mechanisms for the Maintenance of Cellular Fitness. Front Immunol (2014) 5:403. doi: 10.3389/fimmu.2014.00403 25191326 PMC4138502

[58] Hansen TE Johansen T. Following Autophagy Step by Step. BMC Biol (2011) 9(1):39. doi: 10.1186/1741-7007-9-39 21635796 PMC3107173

[59] Ao X Zou L Wu Y. Regulation of Autophagy by the Rab GTPase Network. Cell Death Differ (2014) 21(3):348–58. doi: 10.1038/cdd.2013.187 PMC3921601 24440914

[60] Hammerling BC Najor RH Cortez MQ Shires SE Leon LJ Gonzalez ER. A Rab5 Endosomal Pathway Mediates Parkin-Dependent Mitochondrial Clearance. Nat Commun (2017) 8(1):14050. doi: 10.1038/ncomms14050 28134239 PMC5290275

[61] Martin HL Santoro M Mustafa S Riedel G Forrester JV Teismann P. Evidence for a Role of Adaptive Immune Response in the Disease Pathogenesis of the MPTP Mouse Model of Parkinson’s Disease. Glia (2016) 64(3):386–95. doi: 10.1002/glia.22935 PMC4855685 26511587

[62] Jiang S Gao H Luo Q Wang P Yang X. The Correlation of Lymphocyte Subsets, Natural Killer Cell, and Parkinson’s Disease: A Meta-Analysis. Neurol Sci (2017) 38(8):1373–80. doi: 10.1007/s10072-017-2988-4 28497309

[63] Codolo G Plotegher N Pozzobon T Brucale M Tessari I Bubacco L. Triggering of Inflammasome by Aggregated α-Synuclein, an Inflammatory Response in Synucleinopathies. PloS One (2013) 8(1):e55375. doi: 10.1371/journal.pone.0055375 23383169 PMC3561263

[64] Nagatsu T Sawada M. Inflammatory Process in Parkinson’s Disease: Role for Cytokines. Curr Pharm Des (2005) 11(8):999–1016. doi: 10.2174/1381612053381620 15777250

[65] Ettle B Kuhbandner K Jörg S Hoffmann A Winkler J Linker RA. α-Synuclein Deficiency Promotes Neuroinflammation by Increasing Th1 Cell-Mediated Immune Responses. J Neuroinflamm (2016) 13(1):201. doi: 10.1186/s12974-016-0694-4 PMC5002168 27565429

[66] Giuliani F Goodyer CG Antel JP Yong VW. Vulnerability of Human Neurons to T Cell-Mediated Cytotoxicity. J Immunol (2003) 171(1):368–79. doi: 10.4049/jimmunol.171.1.368 12817020

[67] Prajeeth CK Dittrich-Breiholz O Talbot SR Robert PA Huehn J Stangel M. IFN-γ Producing Th1 Cells Induce Different Transcriptional Profiles in Microglia and Astrocytes. Front Cell Neurosci (2018) 12:352. doi: 10.3389/fncel.2018.00352 30364000 PMC6191492

[68] Appel SH. CD4+ T Cells Mediate Cytotoxicity in Neurodegenerative Diseases. J Clin Invest (2009) 119(1):13–5. doi: 10.1172/jci38096 PMC2613473 19104142

[69] Dombrowski Y O’Hagan T Dittmer M Penalva R Mayoral SR Bankhead P. Regulatory T Cells Promote Myelin Regeneration in the Central Nervous System. Nat Neurosci (2017) 20(5):674–80. doi: 10.1038/nn.4528 PMC5409501 28288125

[70] Lee GR. The Balance of Th17 Versus Treg Cells in Autoimmunity. Int J Mol Sci (2018) 19(3):730. doi: 10.3390/ijms19030730 PMC5877591 29510522

[71] Reynolds AD Stone DK Hutter JA Benner EJ Mosley RL Gendelman HE. Regulatory T Cells Attenuate Th17 Cell-Mediated Nigrostriatal Dopaminergic Neurodegeneration in a Model of Parkinson’s Disease. J Immunol (2010) 184(5):2261–71. doi: 10.4049/jimmunol.0901852 PMC2824790 20118279

[72] Harms AS Cao S Rowse AL Thome AD Li X Mangieri LR. MHCII Is Required for α-Synuclein-Induced Activation of Microglia, CD4 T Cell Proliferation, and Dopaminergic Neurodegeneration. J Neurosci (2013) 33(23):9592–600. doi: 10.1523/jneurosci.5610-12.2013 PMC3903980 23739956

[73] Rostami J Fotaki G Sirois J Mzezewa R Bergström J Essand M. Astrocytes Have the Capacity to Act as Antigen-Presenting Cells in the Parkinson’s Disease Brain. J Neuroinflamm (2020) 17(1):119. doi: 10.1186/s12974-020-01776-7 PMC7164247 32299492

[74] Gericke C Mallone A Engelhardt B Nitsch RM Ferretti MT. Oligomeric Forms of Human Amyloid-Beta(1-42) Inhibit Antigen Presentation. Front Immunol (2020) 11:1029. doi: 10.3389/fimmu.2020.01029 32582162 PMC7290131

[75] Ferretti MT Merlini M Späni C Gericke C Schweizer N Enzmann G. T-Cell Brain Infiltration and Immature Antigen-Presenting Cells in Transgenic Models of Alzheimer’s Disease-Like Cerebral Amyloidosis. Brain Behav Immun (2016) 54:211–25. doi: 10.1016/j.bbi.2016.02.009 26872418

[76] Benner EJ Banerjee R Reynolds AD Sherman S Pisarev VM Tsiperson V. Nitrated Alpha-Synuclein Immunity Accelerates Degeneration of Nigral Dopaminergic Neurons. PloS One (2008) 3(1):e1376. doi: 10.1371/journal.pone.0001376 18167537 PMC2147051

[77] Thome AD Atassi F Wang J Faridar A Zhao W Thonhoff JR. Ex Vivo Expansion of Dysfunctional Regulatory T Lymphocytes Restores Suppressive Function in Parkinson’s Disease. NPJ Parkinson’s Dis (2021) 7(1):41. doi: 10.1038/s41531-021-00188-5 33986285 PMC8119976

[78] Admyre C Grunewald J Thyberg J Gripenbäck S Tornling G Eklund A. Exosomes With Major Histocompatibility Complex Class II and Co-Stimulatory Molecules Are Present in Human BAL Fluid. Eur Respir J (2003) 22(4):578–83. doi: 10.1183/09031936.03.00041703 14582906

[79] Johansson SM Admyre C Scheynius A Gabrielsson S. Different Types of In Vitro Generated Human Monocyte-Derived Dendritic Cells Release Exosomes With Distinct Phenotypes. Immunology (2008) 123(4):491–9. doi: 10.1111/j.1365-2567.2007.02714.x PMC2433319 17949417

[80] Pinnell JR Cui M Tieu K. Exosomes in Parkinson Disease. J Neurochem (2021) 157(3):413–28. doi: 10.1111/jnc.15288 PMC8863192 33372290

[81] Théry C Duban L Segura E Véron P Lantz O Amigorena S. Indirect Activation of Naïve CD4+ T Cells by Dendritic Cell-Derived Exosomes. Nat Immunol (2002) 3(12):1156–62. doi: 10.1038/ni854 12426563

[82] Chutna O Gonçalves S Villar-Piqué A Guerreiro P Marijanovic Z Mendes T. The Small GTPase Rab11 Co-Localizes With α-Synuclein in Intracellular Inclusions and Modulates its Aggregation, Secretion and Toxicity. Hum Mol Genet (2014) 23(25):6732–45. doi: 10.1093/hmg/ddu391 25092884

[83] Gutiérrez-Martínez E Planès R Anselmi G Reynolds M Menezes S Adiko AC. Cross-Presentation of Cell-Associated Antigens by MHC Class I in Dendritic Cell Subsets. Front Immunol (2015) 6:363. doi: 10.3389/fimmu.2015.00363 26236315 PMC4505393

[84] Joffre OP Segura E Savina A Amigorena S. Cross-Presentation by Dendritic Cells. Nat Rev Immunol (2012) 12(8):557–69. doi: 10.1038/nri3254 22790179

[85] Scott CC Vacca F Gruenberg J. Endosome Maturation, Transport and Functions. Semin Cell Dev Biol (2014) 31:2–10. doi: 10.1016/j.semcdb.2014.03.034 24709024

[86] Kleijmeer M Ramm G Schuurhuis D Griffith J Rescigno M Ricciardi-Castagnoli P. Reorganization of Multivesicular Bodies Regulates MHC Class II Antigen Presentation by Dendritic Cells. J Cell Biol (2001) 155(1):53–63. doi: 10.1083/jcb.200103071 11581285 PMC2150788

[87] Münz C. Antigen Processing for MHC Class II Presentation via Autophagy. Front Immunol (2012) 3:9. doi: 10.3389/fimmu.2012.00009 22566895 PMC3342365

[88] You L Mao L Wei J Jin S Yang C Liu H. The Crosstalk Between Autophagic and Endo-/Exosomal Pathways in Antigen Processing for MHC Presentation in Anticancer T Cell Immune Responses. J Hematol Oncol (2017) 10(1):165. doi: 10.1186/s13045-017-0534-8 29058602 PMC5651564

[89] Ganesan D Cai Q. Understanding Amphisomes. Biochem J (2021) 478(10):1959–76. doi: 10.1042/bcj20200917 PMC8935502 34047789

[90] Ojha CR Lapierre J Rodriguez M Dever SM Zadeh MA DeMarino C. Interplay Between Autophagy, Exosomes and HIV-1 Associated Neurological Disorders: New Insights for Diagnosis and Therapeutic Applications. Viruses (2017) 9(7):176. doi: 10.3390/v9070176 PMC5537668 28684681

[91] Birmingham CL Smith AC Bakowski MA Yoshimori T Brumell JH. Autophagy Controls Salmonella Infection in Response to Damage to the Salmonella-Containing Vacuole. J Biol Chem (2006) 281(16):11374–83. doi: 10.1074/jbc.M509157200 16495224

[92] Schmid D Pypaert M Münz C. Antigen-Loading Compartments for Major Histocompatibility Complex Class II Molecules Continuously Receive Input From Autophagosomes. Immunity (2007) 26(1):79–92. doi: 10.1016/j.immuni.2006.10.018 17182262 PMC1805710

[93] Delamarre L Pack M Chang H Mellman I Trombetta ES. Differential Lysosomal Proteolysis in Antigen-Presenting Cells Determines Antigen Fate. Science (2005) 307(5715):1630–4. doi: 10.1126/science.1108003 15761154

[94] Dengjel J Schoor O Fischer R Reich M Kraus M Müller M. Autophagy Promotes MHC Class II Presentation of Peptides From Intracellular Source Proteins. Proc Natl Acad Sci USA (2005) 102(22):7922–7. doi: 10.1073/pnas.0501190102 PMC1142372 15894616

[95] Brazil MI Weiss S Stockinger B. Excessive Degradation of Intracellular Protein in Macrophages Prevents Presentation in the Context of Major Histocompatibility Complex Class II Molecules. Eur J Immunol (1997) 27(6):1506–14. doi: 10.1002/eji.1830270629 9209504

[96] Lanzillotta A Porrini V Bellucci A Benarese M Branca C Parrella E. NF-κb in Innate Neuroprotection and Age-Related Neurodegenerative Diseases. Front Neurol (2015) 6:98. doi: 10.3389/fneur.2015.00098 26042083 PMC4438602

[97] Venkatesha SH Yu H Rajaiah R Tong L Moudgil KD. Celastrus-Derived Celastrol Suppresses Autoimmune Arthritis by Modulating Antigen-Induced Cellular and Humoral Effector Responses. J Biol Chem (2011) 286(17):15138–46. doi: 10.1074/jbc.M111.226365 PMC3083183 21402700

[98] Yang H Liu C Jiang J Wang Y Zhang X. Celastrol Attenuates Multiple Sclerosis and Optic Neuritis in an Experimental Autoimmune Encephalomyelitis Model. Front Pharmacol (2017) 8:44. doi: 10.3389/fphar.2017.00044 28239352 PMC5301323

[99] Harizi H Gualde N. The Impact of Eicosanoids on the Crosstalk Between Innate and Adaptive Immunity: The Key Roles of Dendritic Cells. Tissue Antigens (2005) 65(6):507–14. doi: 10.1111/j.1399-0039.2005.00394.x 15896197

[100] Lee HJ Bazinet RP Rapoport SI Bhattacharjee AK. Brain Arachidonic Acid Cascade Enzymes Are Upregulated in a Rat Model of Unilateral Parkinson Disease. Neurochem Res (2010) 35(4):613–9. doi: 10.1007/s11064-009-0106-6 PMC2836388 19997776

[101] Mayorga LS Colombo MI Lennartz M Brown EJ Rahman KH Weiss R. Inhibition of Endosome Fusion by Phospholipase A2 (PLA2) Inhibitors Points to a Role for PLA2 in Endocytosis. Proc Natl Acad Sci USA (1993) 90(21):10255–9. doi: 10.1073/pnas.90.21.10255 PMC47753 8234286

[102] Zhang C Li A Gao S Zhang X Xiao H. The TIP30 Protein Complex, Arachidonic Acid and Coenzyme A Are Required for Vesicle Membrane Fusion. PloS One (2011) 6(6):e21233. doi: 10.1371/journal.pone.0021233 21731680 PMC3123320

[103] Chock SP Schmauder-Chock EA Cordella-Miele E Miele L Mukherjee AB. The Localization of Phospholipase A2 in the Secretory Granule. Biochem J (1994) 300(Pt 3):619–22. doi: 10.1042/bj3000619 PMC1138211 8010941

[104] Hardman CS Chen YL Salimi M Jarrett R Johnson D Järvinen VJ. CD1a Presentation of Endogenous Antigens by Group 2 Innate Lymphoid Cells. Sci Immunol (2017) 2(18):5918. doi: 10.1126/sciimmunol.aan5918 PMC5826589 29273672

[105] Sarkar C Jones JW Hegdekar N Thayer JA Kumar A Faden AI. PLA2G4A/cPLA2-Mediated Lysosomal Membrane Damage Leads to Inhibition of Autophagy and Neurodegeneration After Brain Trauma. Autophagy (2020) 16(3):466–85. doi: 10.1080/15548627.2019.1628538 PMC6999646 31238788

[106] Deng Z Ma S Zhou H Zang A Fang Y Li T. Tyrosine Phosphatase SHP-2 Mediates C-Type Lectin Receptor-Induced Activation of the Kinase Syk and Anti-Fungal TH17 Responses. Nat Immunol (2015) 16(6):642–52. doi: 10.1038/ni.3155 PMC4439382 25915733

[107] Guo J Huang X Wang H Yang H e0140745. Celastrol Induces Autophagy by Targeting AR/miR-101 in Prostate Cancer Cells. PloS One (2015) 10(10):e0140745. doi: 10.1371/journal.pone.0140745 26473737 PMC4608724

[108] Kissick HT Sanda MG Dunn LK Pellegrini KL On ST Noel JK. Androgens Alter T-Cell Immunity by Inhibiting T-Helper 1 Differentiation. Proc Natl Acad Sci USA (2014) 111(27):9887–92. doi: 10.1073/pnas.1402468111 PMC4103356 24958858

[109] Lai JJ Lai KP Zeng W Chuang KH Altuwaijri S Chang C. Androgen Receptor Influences on Body Defense System via Modulation of Innate and Adaptive Immune Systems: Lessons From Conditional AR Knockout Mice. Am J Pathol (2012) 181(5):1504–12. doi: 10.1016/j.ajpath.2012.07.008 PMC3483803 22959669

[110] McGlinchey RP Lee JC. Cysteine Cathepsins Are Essential in Lysosomal Degradation of α-Synuclein. Proc Natl Acad Sci USA (2015) 112(30):9322–7. doi: 10.1073/pnas.1500937112 PMC4522768 26170293

