# Corrigendum: Celastrol Downmodulates Alpha-Synuclein-Specific T Cell Responses by Mediating Antigen Trafficking in Dendritic Cells

**DOI:** 10.3389/fimmu.2022.907993

**Published:** 2022-05-20

**Authors:** Lam Ng, Xiaohui Wang, Chuanbin Yang, Chengfu Su, Min Li, Allen Ka Loon Cheung

**Affiliations:** ^1^ Department of Biology, Faculty of Science, Hong Kong Baptist University, Kowloon Tong, Hong Kong SAR, China; ^2^ Mr. & Mrs. Ko Chi Ming Center for Parkinson Disease Research, School of Chinese Medicine, Hong Kong Baptist University, Kowloon Tong, Hong Kong SAR, China; ^3^ Department of Geriatrics, Shenzhen People’s Hospital (The Second Clinical Medical College, Jinan University; The First Affiliated Hospital, Southern University of Science and Technology), Shenzhen, China; ^4^ College of Pharmacy, Henan University of Chinese Medicine, Zhengzhou, China

**Keywords:** Celastrol, Parkinson’s Disease, a-synuclein, dendritic cell, CD4+ T cell subsets, endo-lysosomal pathway, autophagy

In the published article, there was an error regarding the affiliation for Min Li. Instead of having affiliations 2,3, the author should only be affiliated to 2.

The authors apologize for this error and state that this does not change the scientific conclusions of the article in any way. The original article has been updated.

## Publisher’s Note

All claims expressed in this article are solely those of the authors and do not necessarily represent those of their affiliated organizations, or those of the publisher, the editors and the reviewers. Any product that may be evaluated in this article, or claim that may be made by its manufacturer, is not guaranteed or endorsed by the publisher.

