# Supplementary material for: Celastrol Downmodulates Alpha-Synuclein-Specific T Cell Responses by Mediating Antigen Trafficking in Dendritic Cells
Source: Front Immunol. 2022 Mar 2;13:833515. doi: 10.3389/fimmu.2022.833515 (PMC8926036; doi:10.3389/fimmu.2022.833515)
Supplement: Supplementary file 1 [file Image_1.pdf]

Supp. Figure 1.

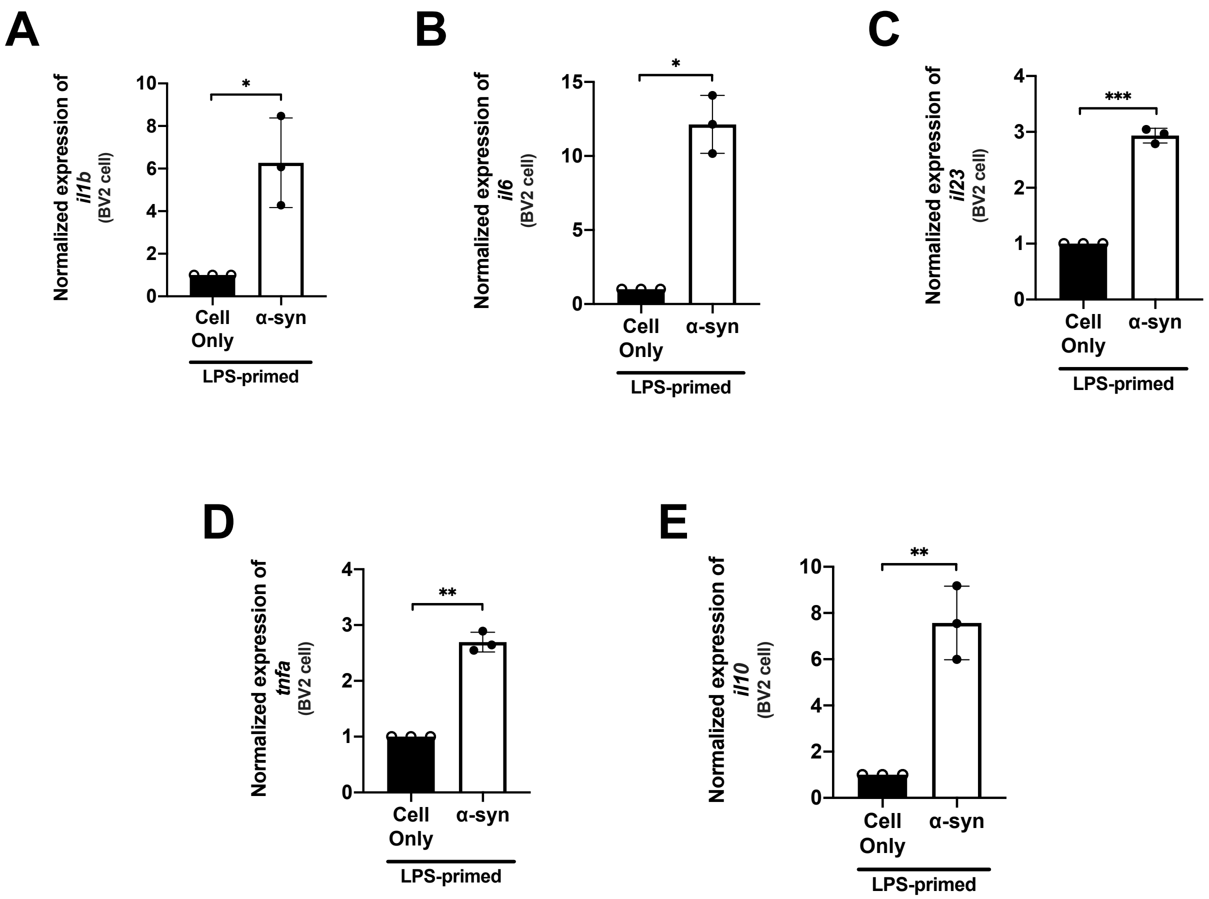

Supp. Figure 1. Inflammatory gene expression of fibrillar α-syn treated BV2. BV2 cells were primed with LPS (100 ng/ml) for 3 h, washed 3 times with PBS and treated with fibrillar α-syn (1 µg/ml) for 3 h. Different cytokine gene expressions were tested by qRT-PCR. Relative gene expressions to β-actin were calculated and normalized to cell only. **(A)** *il1b*, **(B)** *il6*, **(C)** *il23*, **(D)** *tnfa*, **(E)** *il10*. Column graph data represents mean ± SD from 3 individual experiments. Statistical significance was calculated by the Student's *t-test* (one tailed, paired) $^{*}P < 0.05$, $^{**}P < 0.01$, $^{***}P < 0.001$.

Supp. Figure 2.

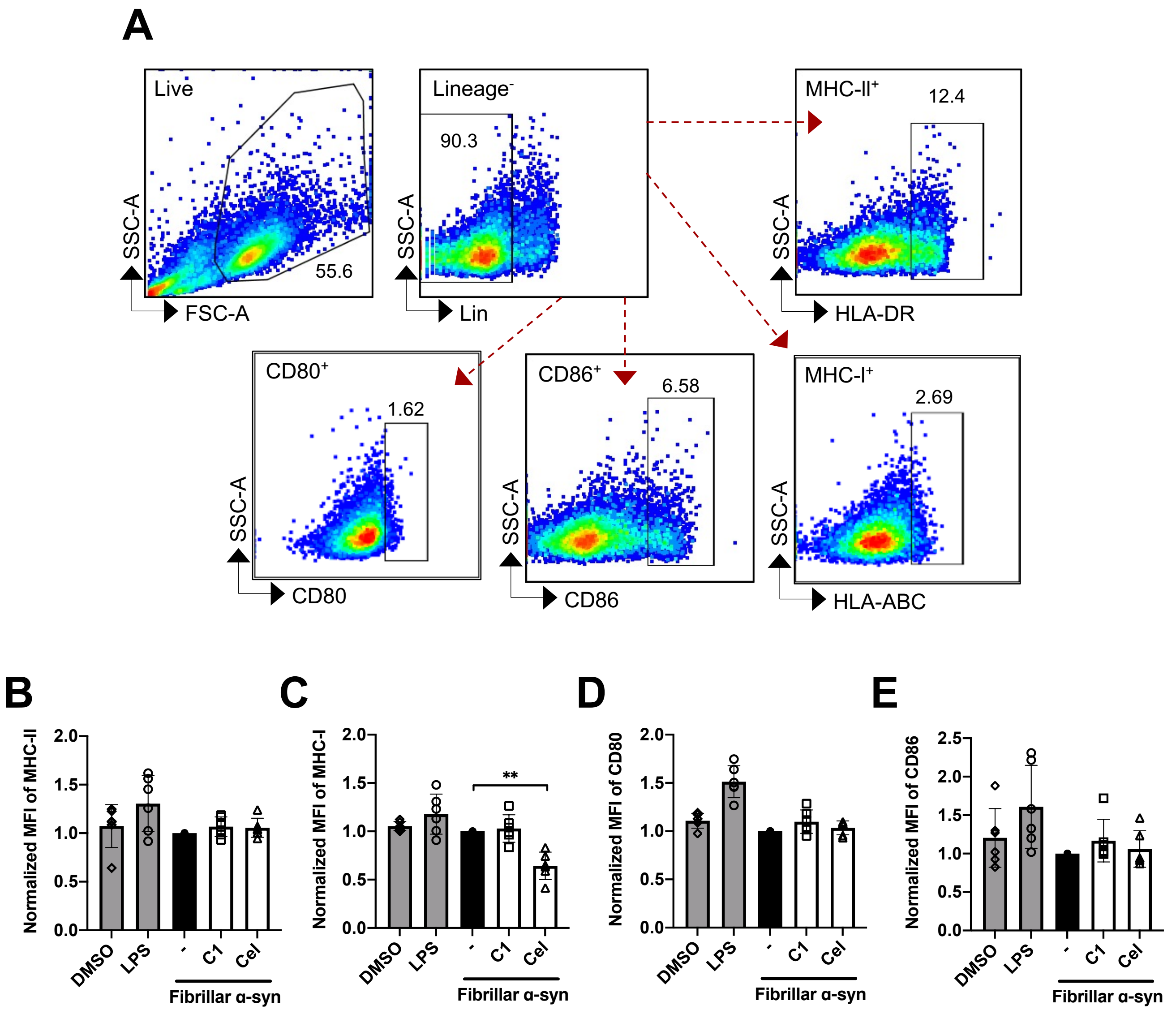

Supp. Figure 2. Flow cytometric analysis of MHC and co-stimulatory molecule surface expression on fibrillar α-syn-treated MoDCs. MoDCs generated from PBMC were pre-treated with C1 (1 μM) or Celastrol (0.25 μM) for 1 h followed by treating with fibrillar α-syn (1 μg/ml) for 24 h. DMSO served as a drug treatment negative control. **(A)** Gating strategy to examine the surface expression of HLA-DR (MHC-II), HLA-ABC (MHC-I), CD80, CD86. **(B-E)** Column graphs of MFI result normalized to α-syn only treatment. Column graph data represents mean ± SD from 6 individual experiments. Statistical significance was calculated by one-way ANOVA and Tukey's multiple comparisons test, $**P < 0.01$.

Supp. Figure 3.

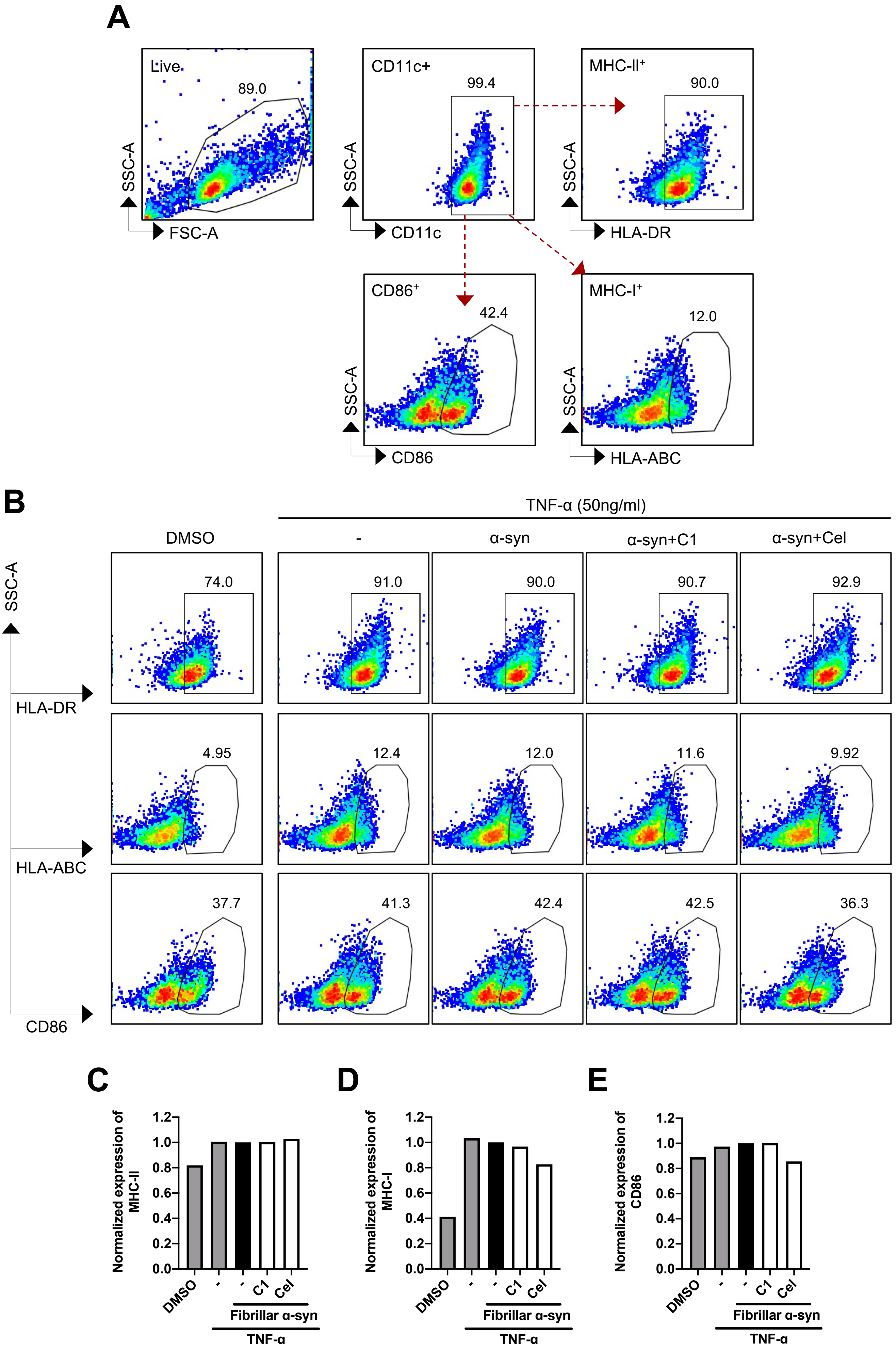

Supp. Figure 3. Gating strategy and flow cytometric analysis of MHC and co-stimulatory molecule surface expression on fibrillar α-syn-treated MoDCs matured with TNF-α. MoDCs generated from PBMC were pre-treated with C1 (1 μM) or Celastrol (0.25 μM) for 1 h followed by matured with TNF-α (50 ng/ml) and treating with fibrillar α-syn (1 μg/ml) for 24 h. Surface expression of HLA-DR (MHC-II), HLA-ABC (MHC-I) and CD86 were assessed by flow cytometry. **(A)** Live cells were gated based on forward and side scatters, followed by gating $CD11c^+$ cells representing MoDCs. $HLA\text{-}DR^+$, $HLA\text{-}ABC^+$ and $CD86^+$ were separately gated afterward. **(B)** Representative flow cytometry analysis plots of HLA-DR (MHC-II), HLA-ABC (MHC-I) and CD86 surface expression of drugs pre-treated, TNF-α matured and α-syn treated MoDCs (numbers indicate percentages) of one experiment. **(C-E)** Column graphs of normalized results of surface expression of different proteins from one experiment.

Supp. Figure 4.

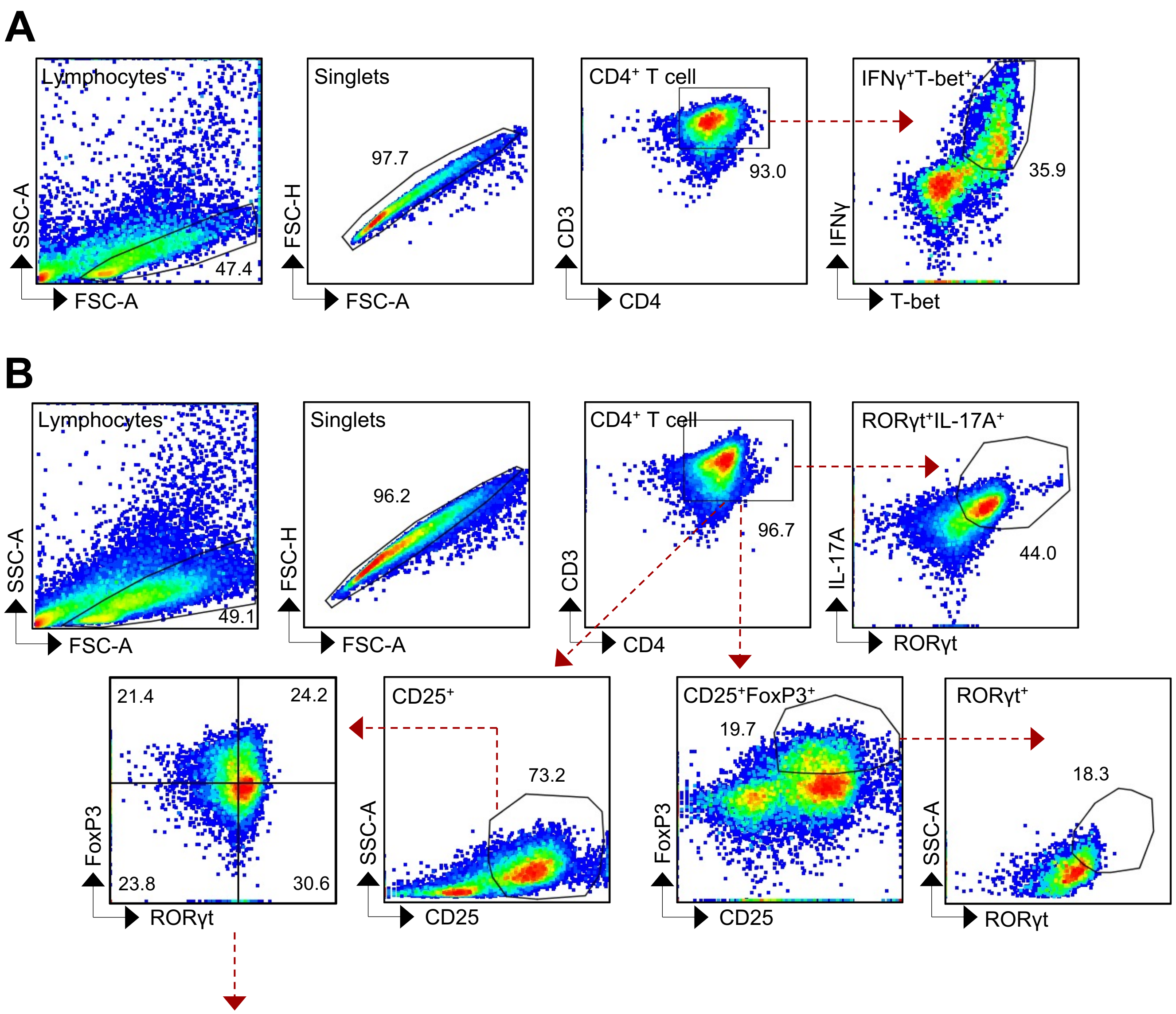

Subpopulations (%) in $CD3^+CD4^+CD25^+$ gated cells (mean ± SD)

RORγt	FoxP3	T cell only	α-syn	α-syn+C1	α-syn+Cel
+	-	2.93±4.92	31.14±3.63	32.77±4.65	7.95±5.45
-	+	6.47±0.64	17.88±3.32	17.58±1.85	30.47±10.09
+	+	2.94±3.62	21.87±2.21	24.70±3.18	15.61±2.15

Supp. Figure 4. Gating strategy for identifying Th1, Th17 and Treg subsets in α-syn-specific $CD4^+$ T cell stimulated by α-syn-pulsed MoDCs co-culture. α-syn-specific $CD4^+$ T cells were co-cultured with C1 or Celastrol pre-treated MoDCs pulsed with α-syn to study the stimulated T cell subsets frequencies. Live cells were gated based on forward and side scatters, followed by singular cells. $CD4^+$ T cells were identified by gating on $CD3^+$ and $CD4^+$ cells and were further gated on **(A)** T-bet$^+$IFNγ$^+$ (Th1) cells; **(B)** RORγt$^+$IL-17A$^+$ (Th17) cells or $CD25^+$FoxP3$^+$ (Treg) cells. RORγt$^+$ cells were also gated from $CD25^+$FoxP3$^+$ (Treg) cells. Alternatively, $CD25^+$ cells were gated from $CD3^+CD4^+$ cells and analyzed for the frequencies of RORγt$^+$FoxP3$^-$, RORγt$^-$ FoxP3$^+$, and FoxP3$^+$RORγt$^+$ subpopulations. The percentage of each subsets from four individual experiment is listed in the table.

Supp. Figure 5.

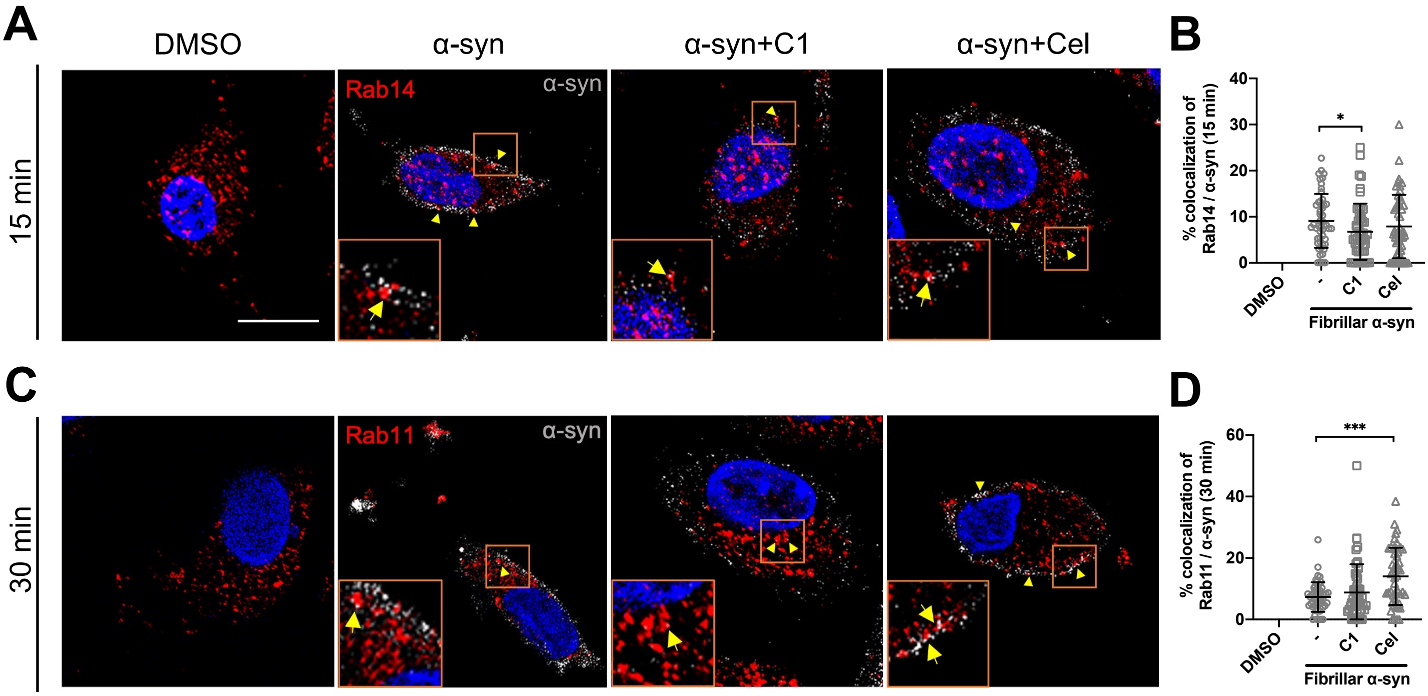

Supp. Figure 5. Colocalization of Rab14 and Rab11 with fibrillar α-syn in MoDCs. MoDCs were pre-treated with C1 (1 µM) or Celastrol (0.25 µM) for 1 h followed by treating with fibrillar α-syn (1 µg/ml) for 15 min and 30 min. Rab14, Rab11 (red) and α-syn (white) were immunostained for the corresponding timepoint with DAPI (blue) and observed under confocal microscope. Representative images showing the colocalization of Rab proteins with α-syn (yellow arrows) under different treatments and dot plots showing the percentage of colocalization. **(A-B)** Colocalization of Rab14 and α-syn at 15 min post α-syn treatment and **(C-D)** colocalization of Rab11 and α-syn at 30 min post α-syn treatment. Images are representative of 50 individual cells. Scale bar: 10 µm. Each dot in the dot plots represents data of a cell and the mean ± SD of 50 individual cells is indicated. Statistical significance was calculated by one-way ANOVA and Tukey's multiple comparisons test, $^{*}P < 0.05$ $^{***}P < 0.001$.

Supp. Figure 6.

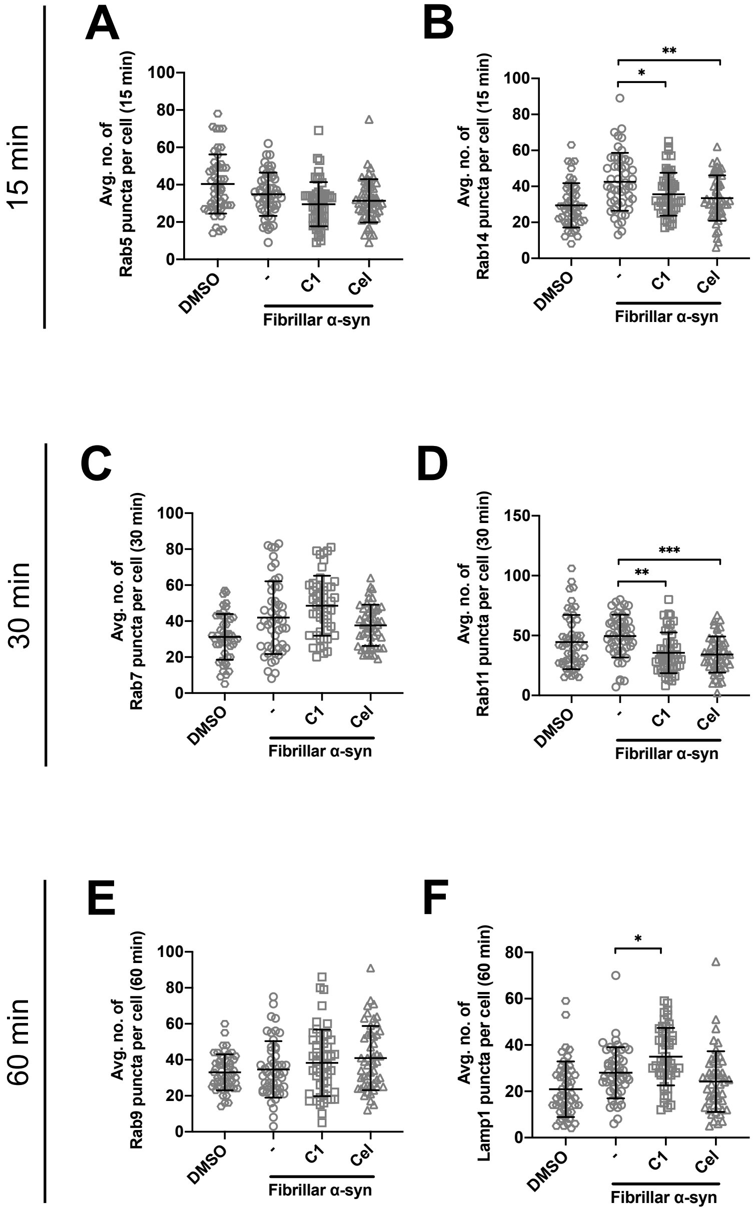

Supp. Figure 6. Analysis of Rab protein and Lamp1 positive puncta per cell in MoDCs following α-syn treatment. MoDCs were pre-treated with C1 (1 µM) or Celastrol (0.25 µM) for 1 h followed by treatment with fibrillar α-syn (1 µg/ml) for 15 min, 30 min and 60 min. Rab proteins and lysosome marker Lamp1 were immunostained for the corresponding timepoint with DAPI and observed under the confocal microscope. The numbers of Rab protein puncta in MoDCs with different treatments were counted at corresponding timepoint and shown as dot plots. The average number of **(A)** Rab5 and **(B)** Rab14 puncta at 15 min, or **(C)** Rab7 and **(D)** Rab11 at 30 min post α-syn treatment, and **(E)** Rab9 and **(F)** Lamp1 at 60 min post α-syn stimulation are shown. Each dot in the dot plots represents data of a cell and the mean ± SD of 50 individual cells is indicated. Statistical significance was calculated by one-way ANOVA and Tukey's multiple comparisons test, **P* < 0.05, ***P* < 0.01, ****P* < 0.001.

Supp. Figure 7.

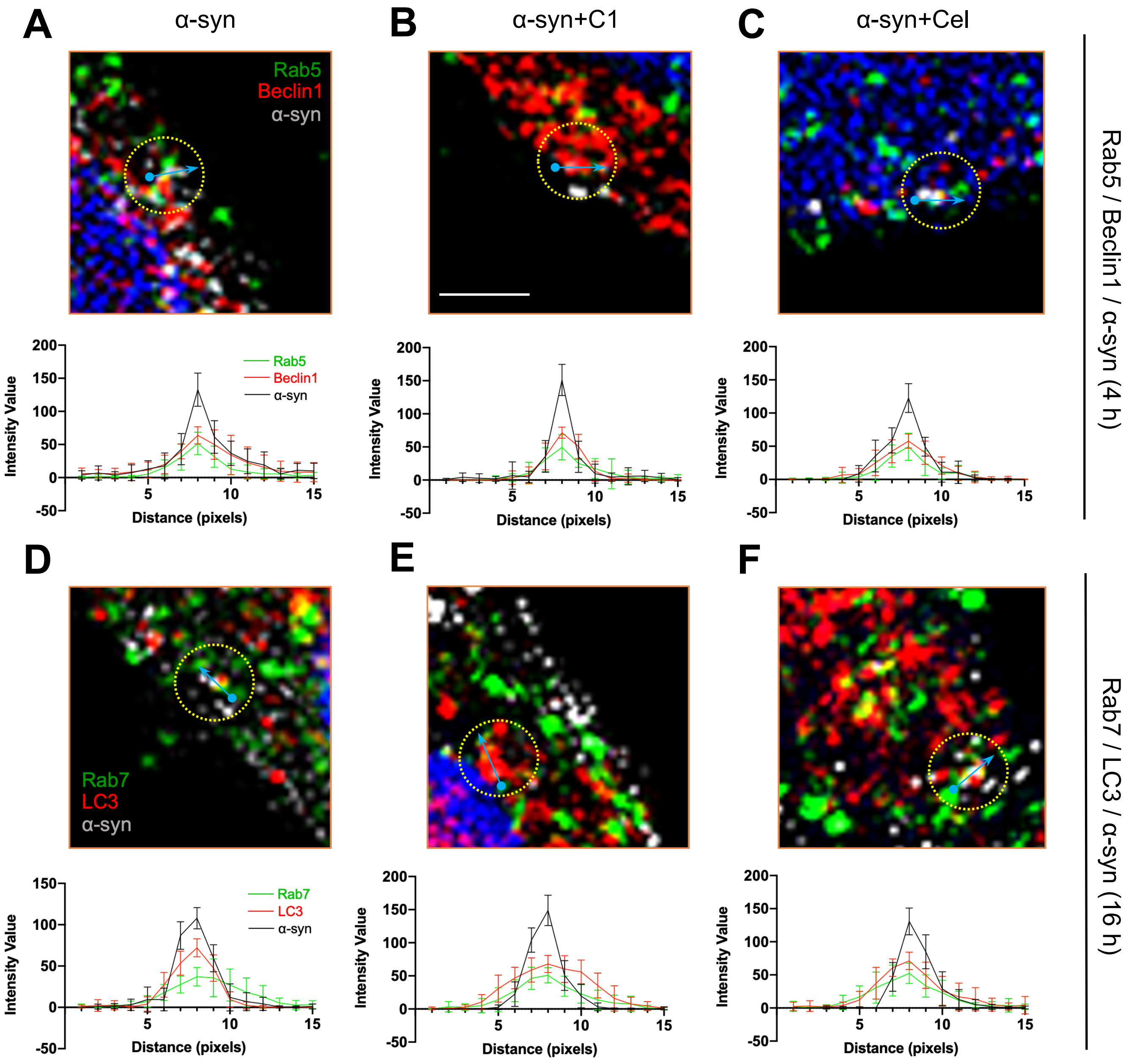

Supp. Figure 7. Colocalization profiles of Rab or autophagic proteins colocalization with α-syn in MoDCs. MoDCs with or without C1 (1 µM) and Celastrol (0.25 µM) pre-treatment were treated with α-syn (1 µg/ml). Images from Figure 5 insets are reproduced here, and the fluorescence signal profiles were analyzed and plotted in a histogram to indicate colocalizations across the distance indicated by the blue arrow equivalent to left to right in the histogram. (**A-C**) Interaction between Rab5 (green line), Beclin1 (red line) and α-syn (black line) are shown, with or without C1 or Celastrol pre-treatment. (**D-F**) Interaction between Rab7 (green line), LC3 (red line) and α-syn (black line) with or without C1 or Celastrol pre-treatment are shown. Images are representative of 50 individual cells. Scale bar: 2 µm. Data represents mean ± SEM of 10 puncta from different individual cells.

Supp. Figure 8.

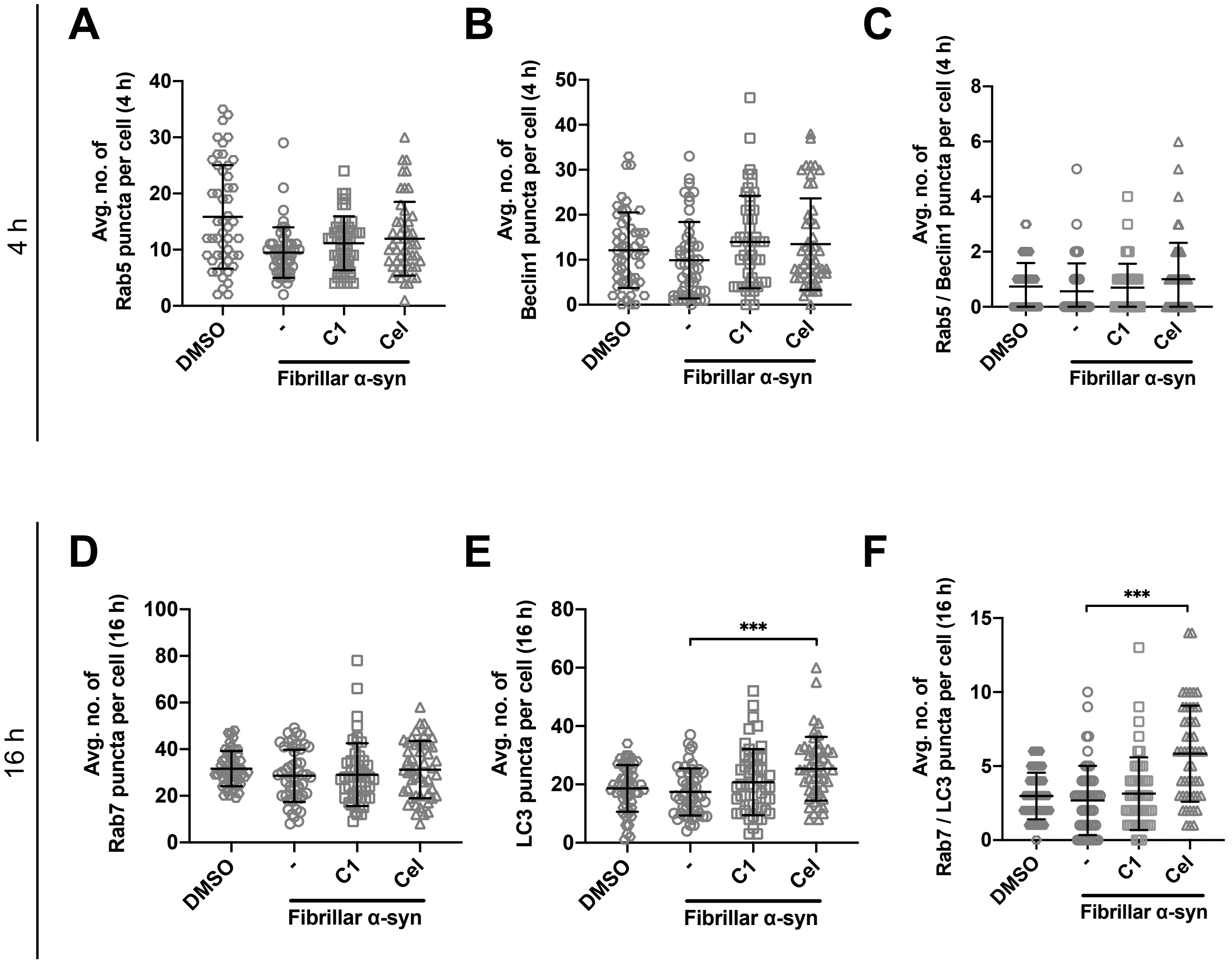

Supp. Figure 8. Analysis of autophagosomes in conjunction to Rab5 and Rab7 puncta in MoDCs after fibrillar α-syn treatment. MoDCs were pre-treated with C1 (1 μM) or Celastrol (0.25 μM) for 1 h followed by fibrillar α-syn (1 μg/ml) treatment for 4 h or 16 h, immunostained and observed under the confocal microscope. The number of Rab5, Rab7, Beclin1 and LC3 positive puncta, and colocalization puncta of the Rab5/Beclin1 and Rab7/LC3 (without α-syn) were counted respectively at each timepoint and shown as dot plots. **(A)** Rab5, **(B)** Beclin1, **(C)** Rab5/Beclin1, **(D)** Rab7, **(E)** LC3 and **(F)** Rab7/LC3 puncta are indicated. Each dot in the dot plots represents data of a cell and the mean ± SD of 50 individual cells is indicated. Statistical significance was calculated by one-way ANOVA and Tukey's multiple comparisons test, ***$P < 0.001$.

Supp. Figure 9.

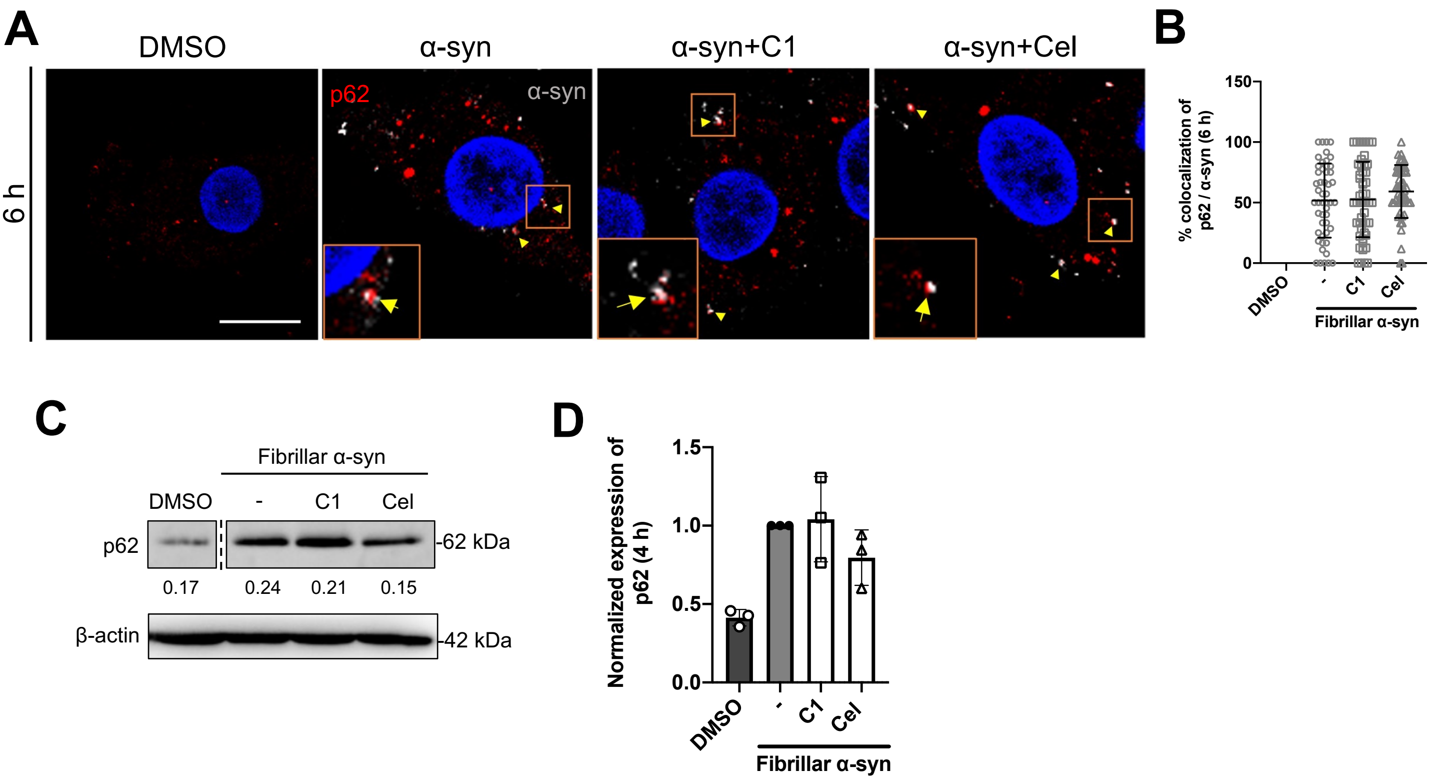

Supp. Figure 9. Expression of p62 and its colocalization with fibrillar α-syn in MoDCs. MoDCs were pre-treated with C1 (1 μM) or Celastrol (0.25 μM) for 1 h followed by treating with fibrillar α-syn (1 μg/ml) for 4 h or 6 h. p62 (red) and α-syn (white) were immunostained with DAPI (blue) and observed under the confocal microscope. Representative images showing the colocalization of p62 with α-syn (yellow arrows) under different treatments and the dot plot showing the percentage of colocalization of each cell. **(A-B)** Colocalization of p62 and α-syn at 6 h post α-syn treatment. Images are representative of 50 individual cells. Scale bar: 10 μm. Each dot in the dot plots represents data of a cell and the mean ± SD of 50 individual cells is indicated. **(C)** The expression of p62 in drug pre-treated MoDCs was determined by Western Blot at 4 h post α-syn treatment. Relative expressions of p62 to β-actin were quantified by ImageJ and indicated on the blots, which were further normalized to α-syn only treatment shown in **(D)**. Column graph data represents mean ± SD from 3 individual experiments. Statistical significance was calculated by one-way ANOVA and Tukey's multiple comparisons test.

Supp. Figure 10.

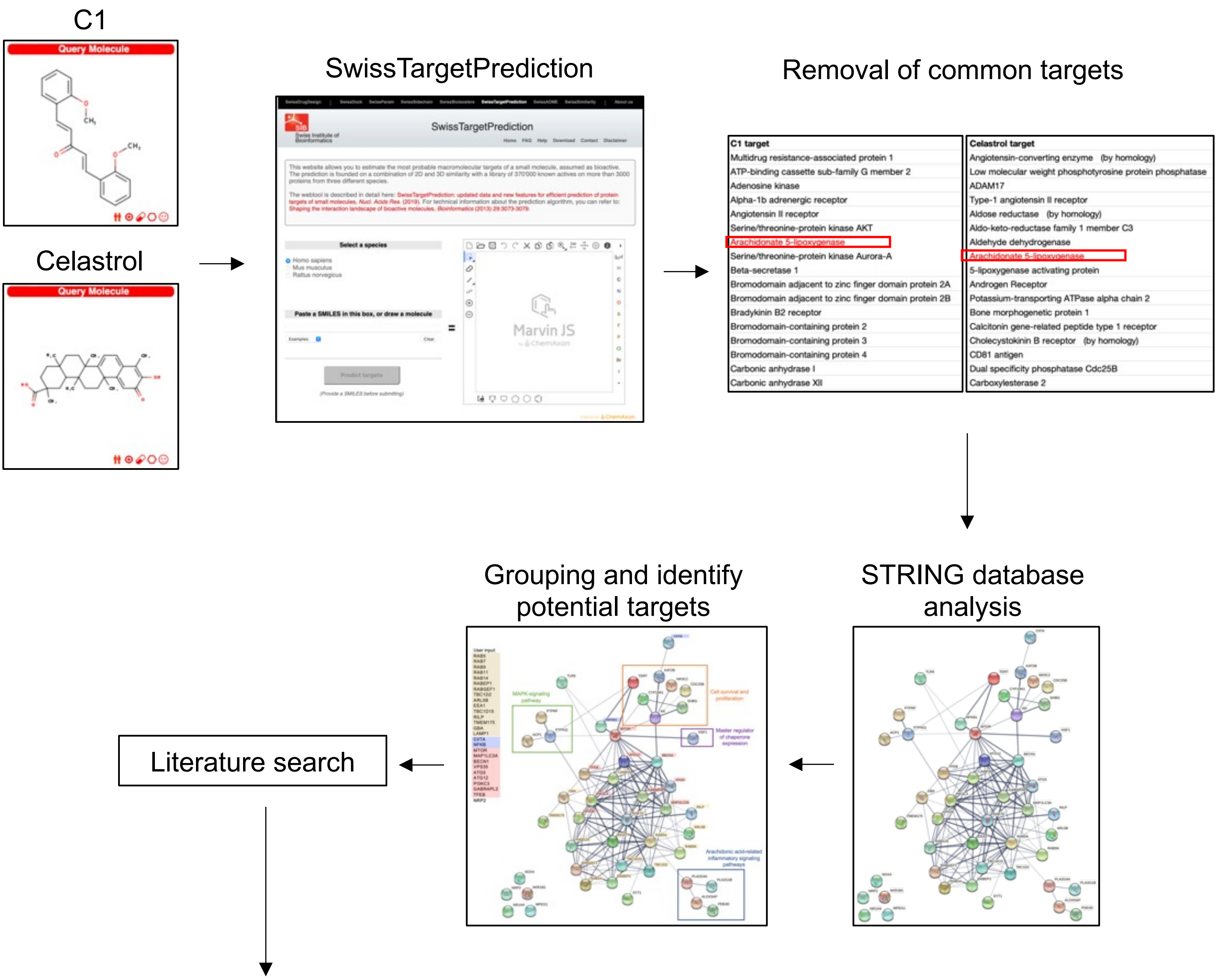

List of potential targets

Celastrol target	Common name	Probability*
Androgen Receptor	AR	0.10063443
Cytosolic phospholipase A2	PLA2G4A	0.10063443
Protein-tyrosine phosphatase 2C	PTPN11	0.64536304

C1 target	Common name	Probability*
Cathepsin (B and K)	CTSB	0.11150187
Cathepsin L	CTSL	0.11150187

Supp. Figure 10. Flow chart of the functional prediction of putative protein targets of C1 or Celastrol. The structure of C1 or Celastrol was inputted into the SwissTargetPrediction tool which generated a list of putative protein targets that Celastrol can possibly interact with. Common protein targets of C1 and Celastrol were removed. Then, the respective list of C1 and Celastrol was inputted into the STRING database separately, together with the known proteins associated with our findings, such as Rab and autophagy-related proteins, and their regulators or effectors, to generate an interactive network. The proteins are manually grouped by their functional similarities. Literature search on each putative protein targets were carried out to identify any possible relationship of them with antigen processing or presentation that could explain the differential effect of C1 and Celastrol in modulating MoDC-mediated T cell responses in PD.

Supp. Figure 11.

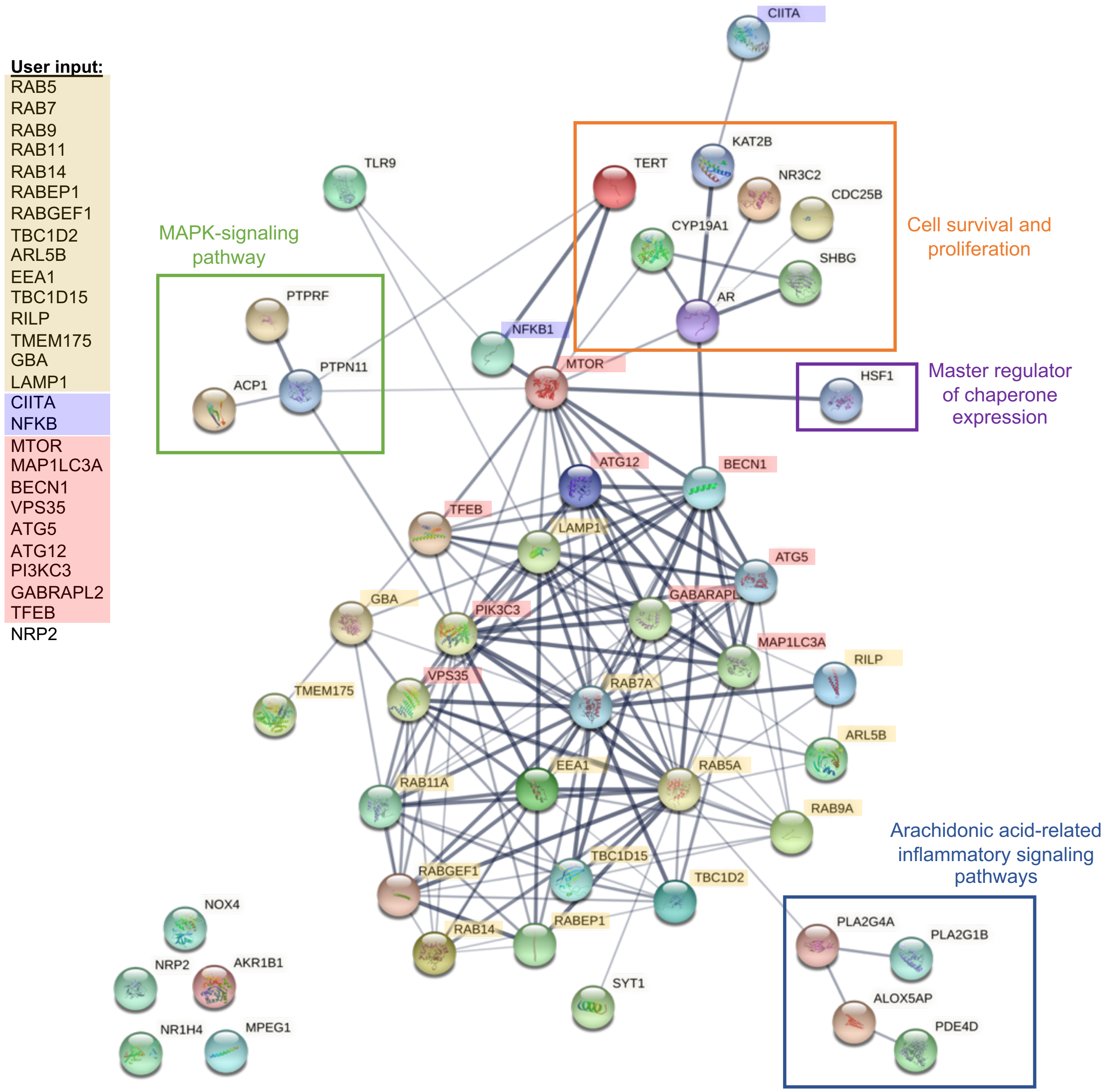

Supp. Figure 11. Functional association analysis of endo-lysosomal or autophagic proteins with putative protein targets of Celastrol. Functional association protein networks using the STRING database were established from the putative protein targets that could interact with Celastrol (SwissTargetPrediction). The lines represent protein-protein associations, and the thickness of the lines represents the strength of data supporting such association. The central cluster contains proteins that are related to endo-lysosomal (names with yellow-brown hue), autophagic pathways (red), and antigen presentation pathway (blue) while other clusters are putative protein targets of Celastrol and are grouped according to their functional similarities.

Supp. Figure 12.

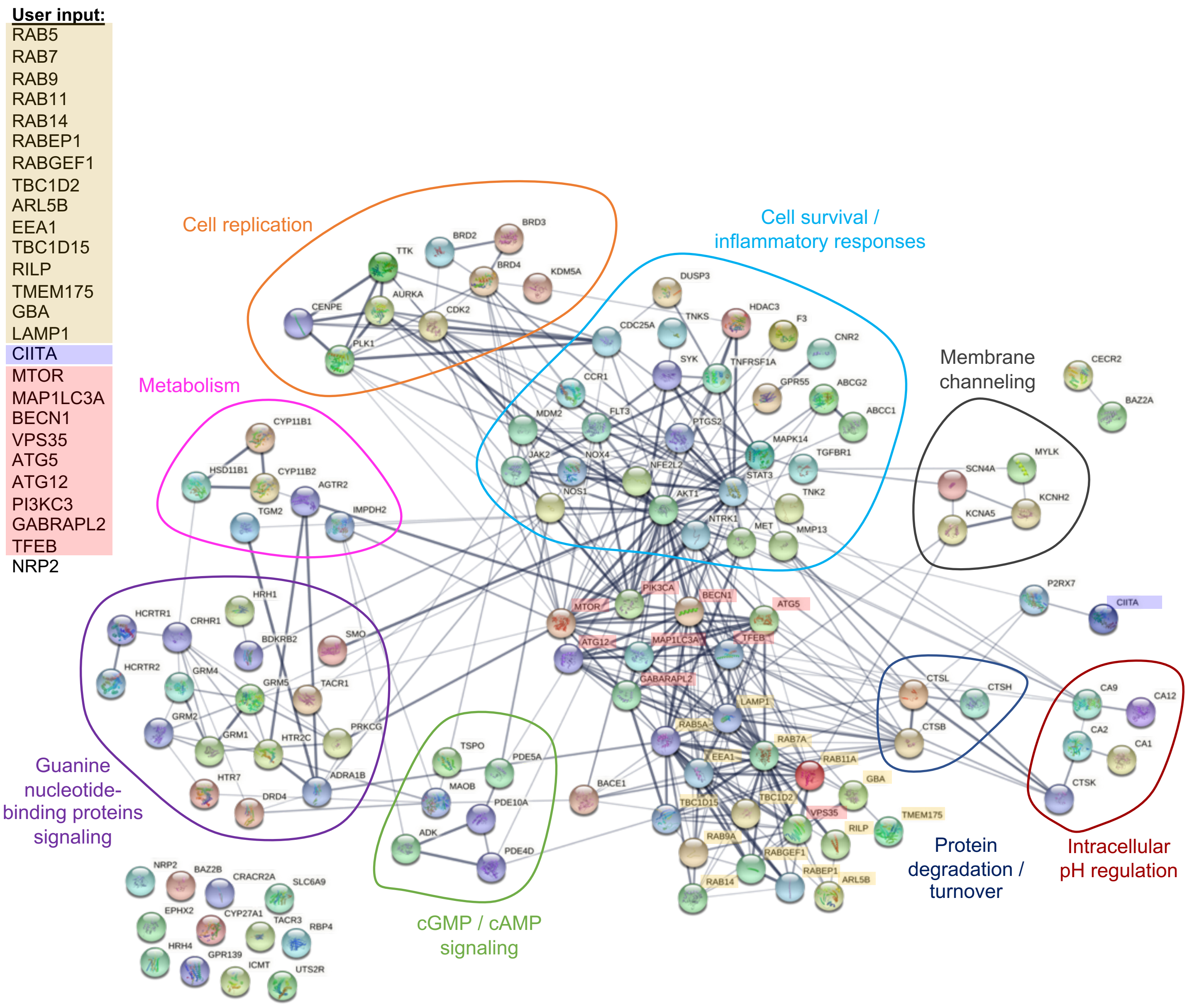

Supp. Figure 12. Functional association analysis of endo-lysosomal or autophagic proteins with putative protein targets of C1. Functional association protein networks using the STRING database were established from the putative protein targets that could interact with C1 (SwissTargetPrediction). The lines represent protein-protein associations, and the thickness of the lines represents the strength of data supporting such association. The central cluster contains proteins that are related to endo-lysosomal (names with yellow-brown hue), autophagic pathways (red), and antigen presentation pathway (blue) while other clusters are putative protein targets of C1 and are grouped according to their functional similarities.

Table S1. Putative protein targets of Celastrol.

SwissTargetPrediction		
**Target**	**Common name**	**Probability**
Toll-like receptor (TLR7/TLR9)	TLR9	1
Protein-tyrosine phosphatase 2C	PTPN11	0.645363044
Aldose reductase (by homology)	AKR1B1	0.402269489
Telomerase reverse transcriptase	TERT	0.1760545
Heat shock factor protein 1	HSF1	0.1760545
Cytochrome P450 19A1	CYP19A1	0.100634432
Phospholipase A2 group 1B	PLA2G1B	0.100634432
Dual specificity phosphatase Cdc25B	CDC25B	0.100634432
Receptor-type tyrosine-protein phosphatase F (LAR)	PTPRF	0.100634432
Low molecular weight phosphotyrosine protein phosphatase	ACP1	0.100634432
Phosphodiesterase 4D	PDE4D	0.100634432
Testis-specific androgen-binding protein	SHBG	0.100634432
Macrophage-expressed gene 1 protein	MPEG1	0.100634432
Histone acetyltransferase PCAF	KAT2B	0.100634432
5-lipoxygenase activating protein	ALOX5AP	0.100634432
Mineralocorticoid receptor	NR3C2	0.100634432
Cytosolic phospholipase A2	PLA2G4A	0.100634432
Bile acid receptor FXR	NR1H4	0.100634432
Androgen Receptor	AR	0.100634432

Only those targets predicted with possibility > 0 were shown.

Table S2. Putative protein targets of C1.

SwissTargetPrediction		
**Target**	**Common name**	**Probability**
Multidrug resistance-associated protein 1	ABCC1	0.111501865
ATP-binding cassette sub-family G member 2	ABCG2	0.111501865
Adenosine kinase	ADK	0.111501865
Alpha-1b adrenergic receptor	ADRA1B	0.111501865
Angiotensin II receptor	AGTR2	0.111501865
Serine/threonine-protein kinase AKT	AKT1	0.111501865
Serine/threonine-protein kinase Aurora-A	AURKA	0.111501865
Beta-secretase 1	BACE1	0.111501865
Bromodomain adjacent to zinc finger domain protein 2A	BAZ2A	0.111501865
Bromodomain adjacent to zinc finger domain protein 2B	BAZ2B	0.111501865
Bradykinin B2 receptor	BDKRB2	0.111501865
Bromodomain-containing protein 2	BRD2	0.111501865
Bromodomain-containing protein 3	BRD3	0.111501865
Bromodomain-containing protein 4	BRD4	0.111501865
Carbonic anhydrase I	CA1	0.111501865
Carbonic anhydrase XII	CA12	0.111501865
Carbonic anhydrase II	CA2	0.111501865
Carbonic anhydrase IX	CA9	0.111501865
C-C chemokine receptor type 1	CCR1	0.111501865
Dual specificity phosphatase Cdc25A	CDC25A	0.111501865
Cyclin-dependent kinase 2	CDK2	0.111501865
Cyclin-dependent kinase 2/cyclin A	CDK2 CCNA1 CCNA2	0.111501865
CDK9/cyclin T1	CDK9 CCNT1	0.111501865
Cat eye syndrome critical region protein 2	CECR2	0.111501865
Centromere-associated protein E	CENPE	0.111501865
Cannabinoid receptor 2	CNR2	0.111501865
EF-hand calcium-binding domain-containing protein 4B	CRACR2A	0.111501865
Corticotropin releasing factor receptor 1	CRHR1	0.111501865
Cathepsin (B and K)	CTSB	0.111501865
Cathepsin (H and K)	CTSH	0.111501865
Cathepsin K	CTSK	0.111501865
Cathepsin L	CTSL	0.111501865
Cytochrome P450 11B1	CYP11B1	0.111501865
Cytochrome P450 11B2	CYP11B2	0.111501865
Sterol 26-hydroxylase, mitochondrial	CYP27A1	0.111501865
Dopamine D4 receptor	DRD4	0.111501865
Dual specificity protein phosphatase 3	DUSP3	0.111501865
Epoxide hydratase	EPHX2	0.111501865
Coagulation factor VII/tissue factor	F3	0.111501865
Tyrosine-protein kinase receptor FLT3	FLT3	0.111501865
Protein farnesyltransferase	FNTA FNTB	0.111501865
Probable G-protein coupled receptor 139	GPR139	0.111501865
G-protein coupled receptor 55	GPR55	0.111501865
Metabotropic glutamate receptor 1	GRM1	0.111501865
Metabotropic glutamate receptor 2	GRM2	0.111501865
Metabotropic glutamate receptor 4	GRM4	0.111501865
Metabotropic glutamate receptor 5	GRM5	0.111501865
Orexin receptor 1	HCRTR1	0.111501865

SwissTargetPrediction		
**Target**	**Common name**	**Probability**
Orexin receptor 2	HCRTR2	0.111501865
Histone deacetylase 3	HDAC3	0.111501865
Histamine H1 receptor	HRH1	0.111501865
Histamine H4 receptor	HRH4	0.111501865
11-beta-hydroxysteroid dehydrogenase 1	HSD11B1	0.111501865
Serotonin 2c (5-HT2c) receptor	HTR2C	0.111501865
Serotonin 7 (5-HT7) receptor	HTR7	0.111501865
Isoprenylcysteine carboxyl methyltransferase	ICMT	0.111501865
Inosine-5'-monophosphate dehydrogenase 2	IMPDH2	0.111501865
Tyrosine-protein kinase JAK2	JAK2	0.111501865
Voltage-gated potassium channel subunit Kv1.5	KCNA5	0.111501865
HERG	KCNH2	0.111501865
Lysine-specific demethylase 5A	KDM5A	0.111501865
Monoamine oxidase B	MAOB	0.111501865
MAP kinase p38 alpha	MAPK14	0.111501865
p53-binding protein Mdm-2	MDM2	0.111501865
Hepatocyte growth factor receptor	MET	0.111501865
Matrix metalloproteinase 13	MMP13	0.111501865
Serine/threonine-protein kinase mTOR (by homology)	MTOR	0.111501865
Myosin light chain kinase, smooth muscle	MYLK	0.111501865
Nuclear factor erythroid 2-related factor 2	NFE2L2	0.111501865
Nitric-oxide synthase, brain	NOS1	0.111501865
Nerve growth factor receptor Trk-A	NTRK1	0.111501865
P2X purinoceptor 7	P2RX7	0.111501865
Phosphodiesterase 10A	PDE10A	0.111501865
Phosphodiesterase 4D	PDE4D	0.111501865
Phosphodiesterase 5A	PDE5A	0.111501865
PI3-kinase p110-alpha subunit	PIK3CA	0.111501865
Serine/threonine-protein kinase PLK1	PLK1	0.111501865
Protein kinase C gamma (by homology)	PRKCG	0.111501865
Gamma-secretase	PSEN2 PSENEN NCSTN APH1A PSEN1 APH1B	0.111501865
Cyclooxygenase-2	PTGS2	0.111501865
Plasma retinol-binding protein	RBP4	0.111501865
Sodium channel protein type IV alpha subunit	SCN4A	0.111501865
Glycine transporter 1	SLC6A9	0.111501865
Smoothened homolog	SMO	0.111501865
Signal transducer and activator of transcription 3	STAT3	0.111501865
Tyrosine-protein kinase SYK	SYK	0.111501865
Neurokinin 1 receptor	TACR1	0.111501865
Neurokinin 3 receptor	TACR3	0.111501865
TGF-beta receptor type I	TGFBR1	0.111501865
Protein-glutamine gamma-glutamyltransferase	TGM2	0.111501865
Tumor necrosis factor receptor R1	TNFRSF1A	0.111501865
Tyrosine kinase non-receptor protein 2	TNK2	0.111501865
Tankyrase-1	TNKS	0.111501865
Translocator protein (by homology)	TSPO	0.111501865
Dual specificity protein kinase TTK	TTK	0.111501865
Urotensin II receptor	UTS2R	0.111501865

Only those targets predicted with possibility > 0 were shown.
