# Supplementary material for: Celastrol Downmodulates Alpha-Synuclein-Specific T Cell Responses by Mediating Antigen Trafficking in Dendritic Cells
Source: Front Immunol. 2022 Mar 2;13:833515. doi: 10.3389/fimmu.2022.833515 (PMC8926036; doi:10.3389/fimmu.2022.833515)

Original photo of immunoblot for Figure 6 A – Beclin1

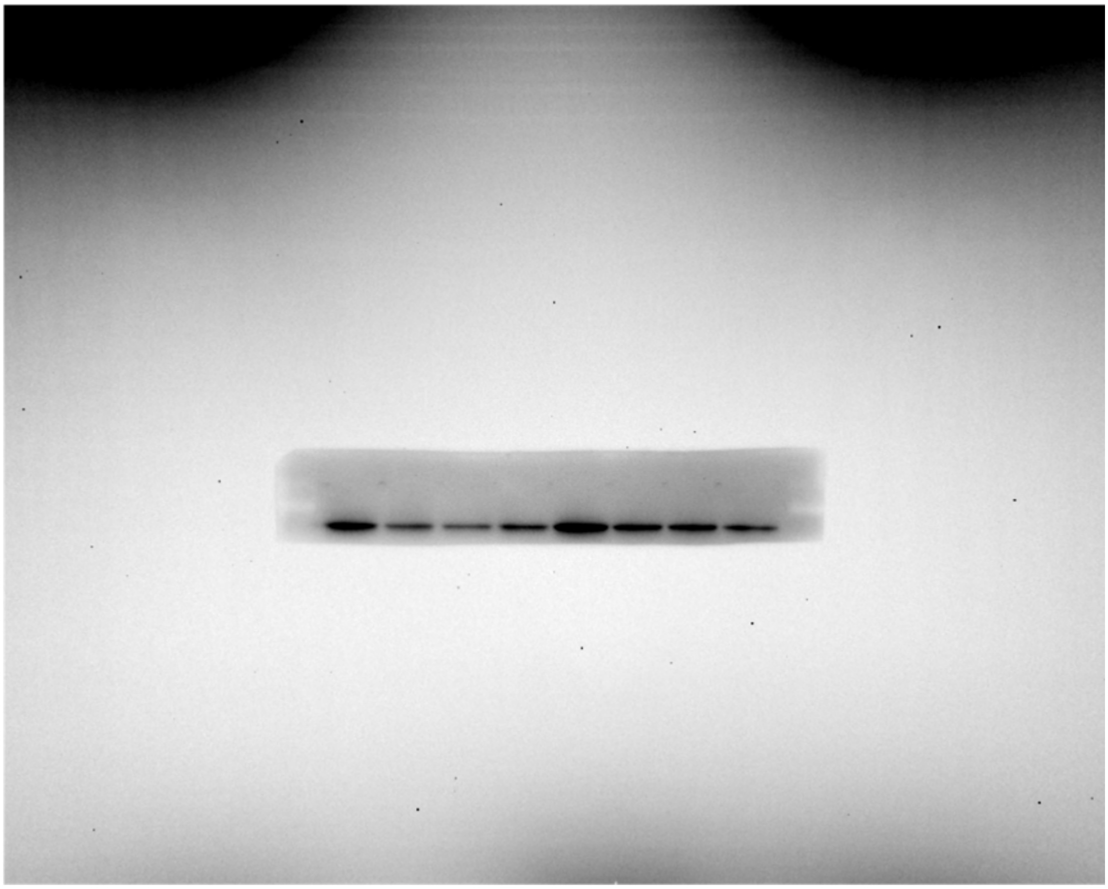

Original photo of immunoblot for Figure 6 A – β-actin

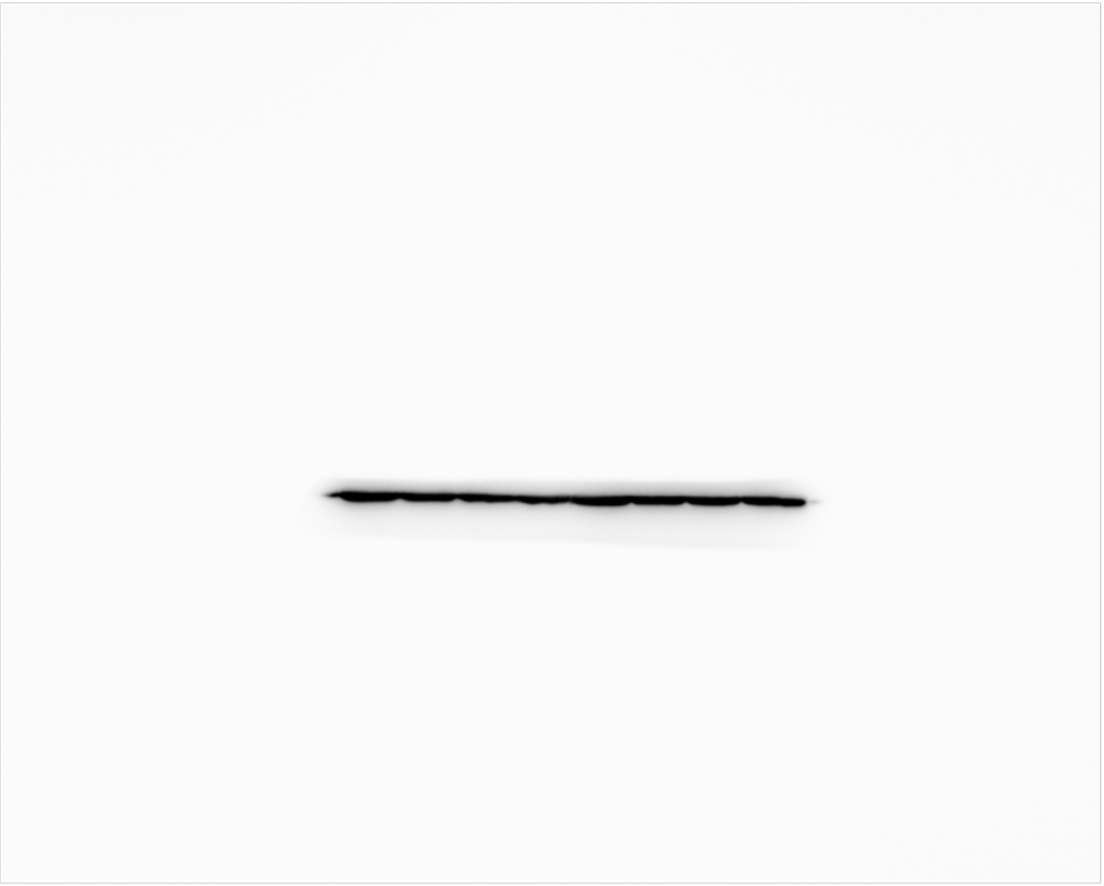

Original photo of immunoblot for Figure 6 C – LC3-I and LC3-II

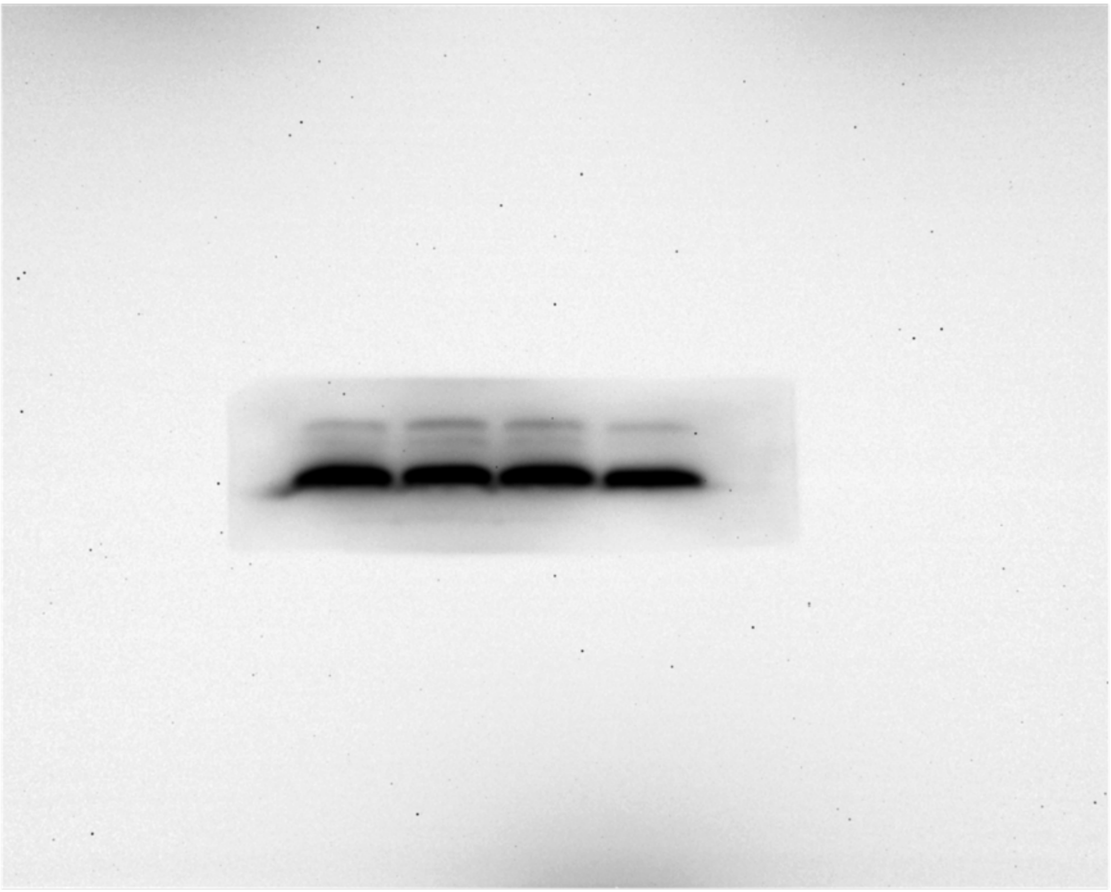

Original photo of immunoblot for Figure 6 C – β-actin

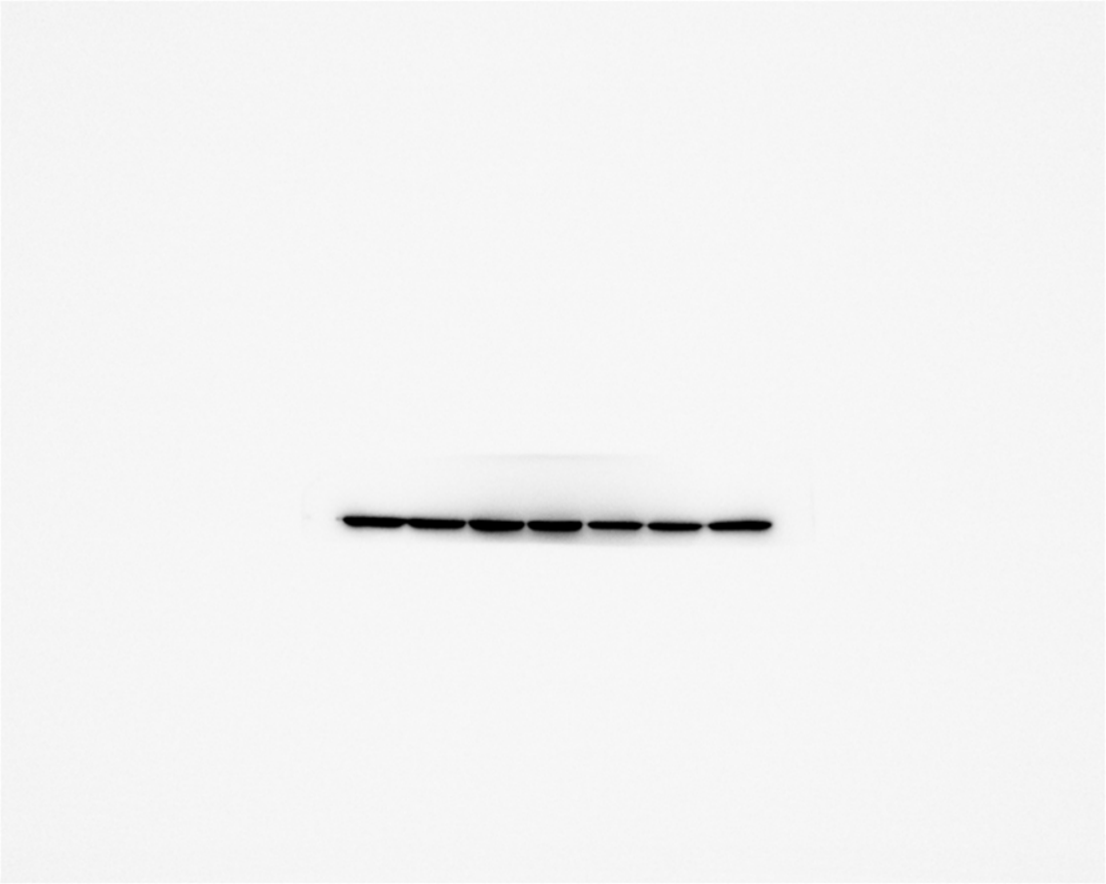

Original photo of immunoblot for Figure 6 G – Rab5-GTP

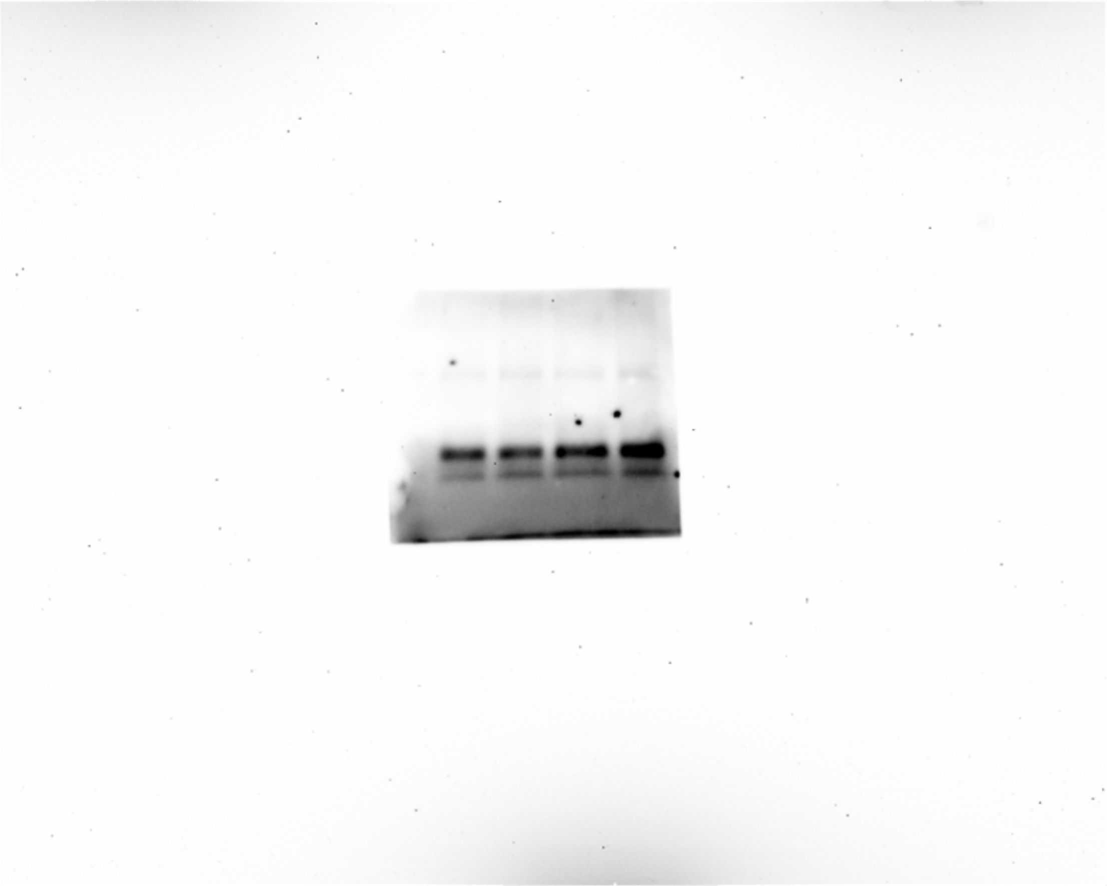

Original photo of immunoblot for Figure 6 G – Input

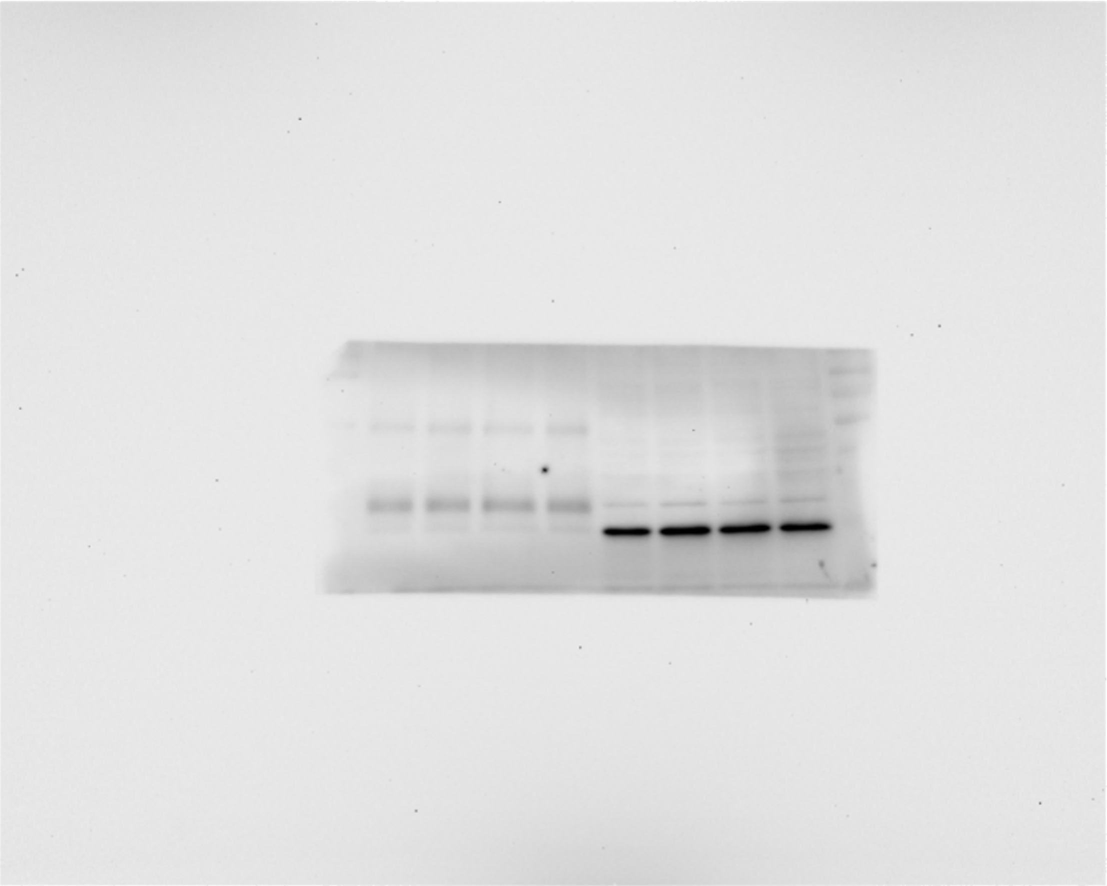

Original photo of immunoblot for Figure 6 H – Rab7-GTP

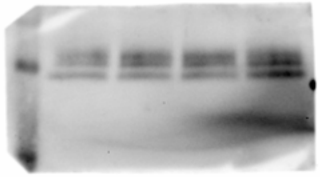

Original photo of immunoblot for Figure 6 H – Input

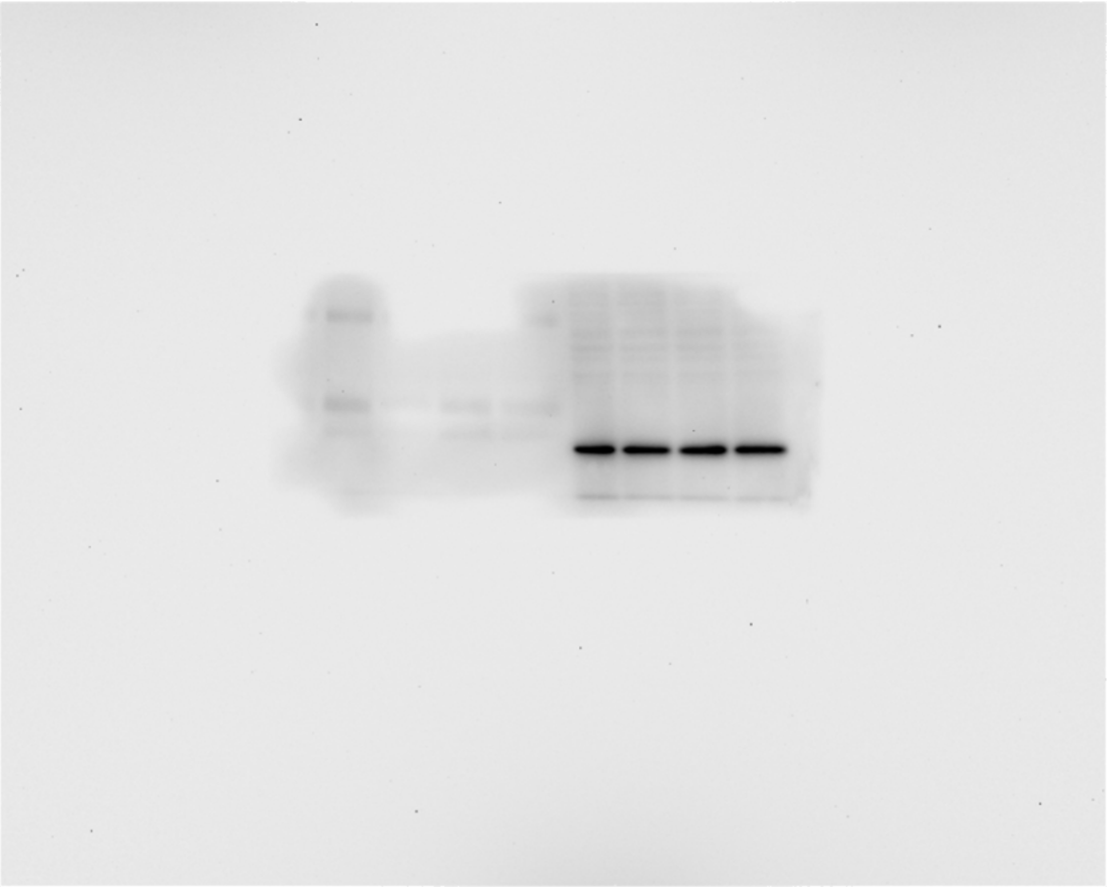

Original photo of immunoblot for Supp. Figure 9 C – p62

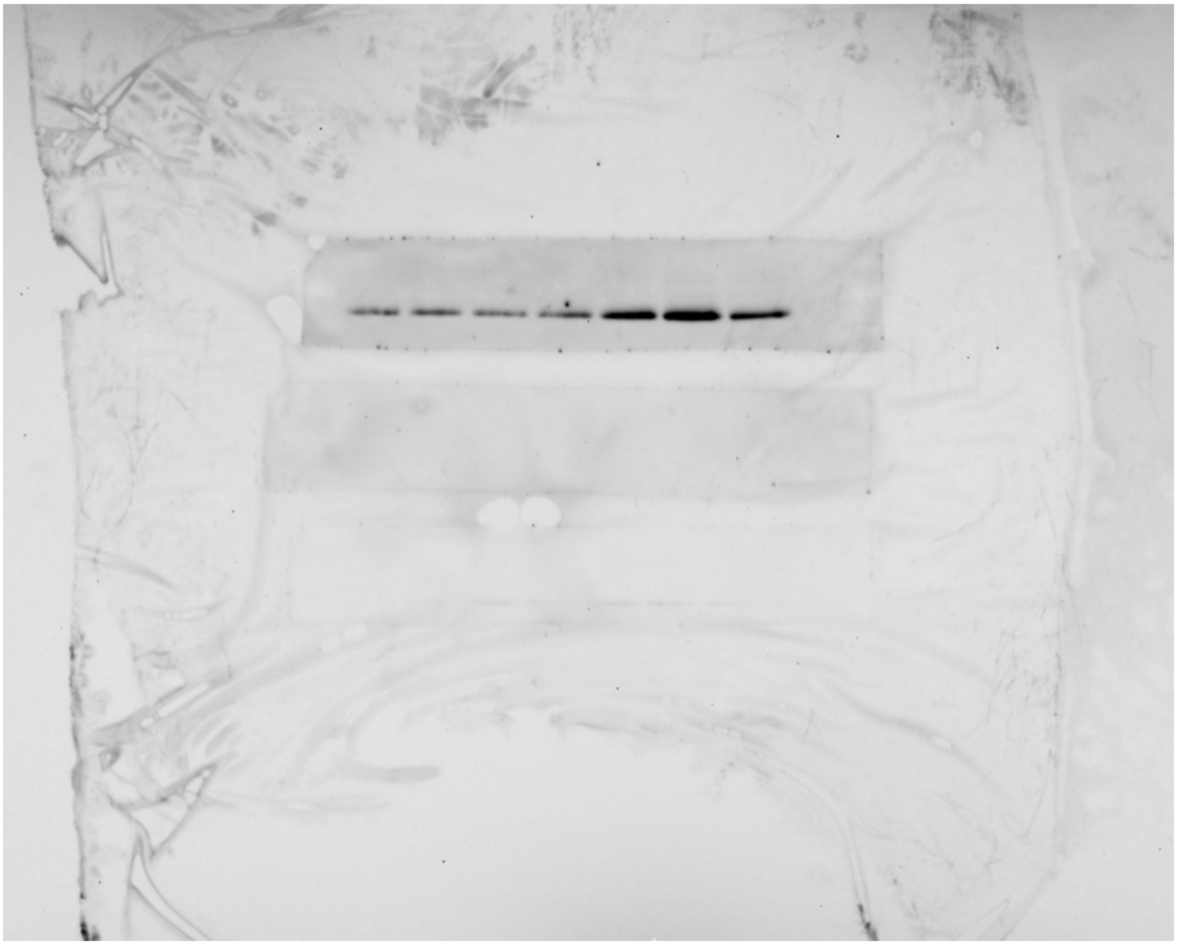

Supplement: Supplementary file 2 [file Image_2.pdf]
